# Repurposing the mucolytic agent ambroxol for treatment of sub-acute and chronic ischaemic stroke

**DOI:** 10.1093/braincomms/fcad099

**Published:** 2023-03-29

**Authors:** Kristin Patzwaldt, Georgy Berezhnoy, Tudor Ionescu, Linda Schramm, Yi Wang, Miriam Owczorz, Eduardo Calderón, Sven Poli, Lina M Serna Higuita, Irene Gonzalez-Menendez, Leticia Quintanilla-Martinez, Kristina Herfert, Bernd Pichler, Christoph Trautwein, Salvador Castaneda-Vega

**Affiliations:** Werner Siemens Imaging Center, Department of Preclinical Imaging and Radiopharmacy, Eberhard Karls University Tuebingen, Tuebingen 72076, Germany; Werner Siemens Imaging Center, Department of Preclinical Imaging and Radiopharmacy, Eberhard Karls University Tuebingen, Tuebingen 72076, Germany; Werner Siemens Imaging Center, Department of Preclinical Imaging and Radiopharmacy, Eberhard Karls University Tuebingen, Tuebingen 72076, Germany; Werner Siemens Imaging Center, Department of Preclinical Imaging and Radiopharmacy, Eberhard Karls University Tuebingen, Tuebingen 72076, Germany; Hertie Institute for Clinical Brain Research, Department for Neurology, University Hospital Tuebingen, Tuebingen 72076, Germany; Werner Siemens Imaging Center, Department of Preclinical Imaging and Radiopharmacy, Eberhard Karls University Tuebingen, Tuebingen 72076, Germany; Department of Nuclear Medicine and Clinical Molecular Imaging, University Hospital Tuebingen, Tuebingen 72076, Germany; Hertie Institute for Clinical Brain Research, Department for Neurology, University Hospital Tuebingen, Tuebingen 72076, Germany; Institute for Clinical Epidemiology and Applied Biostatistics, University Hospital Tuebingen, Tuebingen 72076, Germany; Institute of Pathology and Neuropathology, Comprehensive Cancer Center, Eberhard Karls University, Tuebingen 72076, Germany; Cluster of Excellence iFIT (EXC 2180) ‘Image-Guided and Functionally Instructed Tumor Therapies’, Eberhard Karls University Tuebingen, Tuebingen 72076, Germany; Institute of Pathology and Neuropathology, Comprehensive Cancer Center, Eberhard Karls University, Tuebingen 72076, Germany; Cluster of Excellence iFIT (EXC 2180) ‘Image-Guided and Functionally Instructed Tumor Therapies’, Eberhard Karls University Tuebingen, Tuebingen 72076, Germany; Werner Siemens Imaging Center, Department of Preclinical Imaging and Radiopharmacy, Eberhard Karls University Tuebingen, Tuebingen 72076, Germany; Werner Siemens Imaging Center, Department of Preclinical Imaging and Radiopharmacy, Eberhard Karls University Tuebingen, Tuebingen 72076, Germany; Cluster of Excellence iFIT (EXC 2180) ‘Image-Guided and Functionally Instructed Tumor Therapies’, Eberhard Karls University Tuebingen, Tuebingen 72076, Germany; Werner Siemens Imaging Center, Department of Preclinical Imaging and Radiopharmacy, Eberhard Karls University Tuebingen, Tuebingen 72076, Germany; Werner Siemens Imaging Center, Department of Preclinical Imaging and Radiopharmacy, Eberhard Karls University Tuebingen, Tuebingen 72076, Germany; Department of Nuclear Medicine and Clinical Molecular Imaging, University Hospital Tuebingen, Tuebingen 72076, Germany

**Keywords:** stroke, neuroprotection, neuroimaging, drug repurposing, ambroxol

## Abstract

Ambroxol is a well-known mucolytic expectorant, which has gained much attention in amyotrophic lateral sclerosis, Parkinson’s and Gaucher’s disease. A specific focus has been placed on ambroxol’s glucocerebrosidase-stimulating activity, on grounds that the point mutation of the *gba1* gene, which codes for this enzyme, is a risk factor for developing Parkinson’s disease. However, ambroxol has been attributed other characteristics, such as the potent inhibition of sodium channels, modification of calcium homeostasis, anti-inflammatory effects and modifications of oxygen radical scavengers. We hypothesized that ambroxol could have a direct impact on neuronal rescue if administered directly after ischaemic stroke induction. We longitudinally evaluated 53 rats using magnetic resonance imaging to examine stroke volume, oedema, white matter integrity, resting state functional MRI and behaviour for 1 month after ischemic stroke onset. For closer mechanistic insights, we evaluated tissue metabolomics of different brain regions in a subgroup of animals using *ex vivo* nuclear magnetic resonance spectroscopy.

Ambroxol-treated animals presented reduced stroke volumes, reduced cytotoxic oedema, reduced white matter degeneration, reduced necrosis, improved behavioural outcomes and complex changes in functional brain connectivity. Nuclear magnetic resonance spectroscopy tissue metabolomic data at 24 h post-stroke proposes several metabolites that are capable of minimizing post-ischaemic damage and that presented prominent shifts during ambroxol treatment in comparison to controls. Taking everything together, we propose that ambroxol catalyzes recovery in energy metabolism, cellular homeostasis, membrane repair mechanisms and redox balance. One week of ambroxol administration following stroke onset reduced ischaemic stroke severity and improved functional outcome in the subacute phase followed by reduced necrosis in the chronic stroke phase.

## Teaser

Patzwaldt *et al*. demonstrate that ambroxol, the over-the-counter mucolytic agent, induces neuroprotection after ischaemic stroke in rats. Advanced MRI techniques, behavioural analysis and study of metabolomics provide complementary multimodal evidence that ambroxol improves the outcome of stroke lesions and brain function.

## Graphical Abstract

**Graphical abstract:**
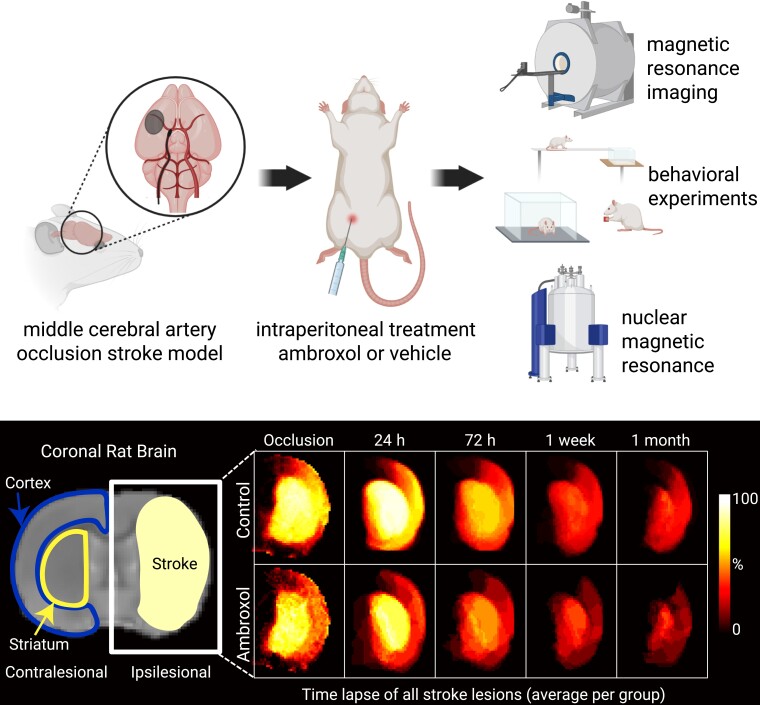

## Introduction

Ischemic stroke (IS) is a sudden and life-threatening disease with suboptimal treatment options. The American Heart Association estimates an additional 3.4 million patients developing stroke by 2030 in the United States.^1^ However, these predictions may worsen since the global pandemic of corona virus disease 2019 further increased the incidence of IS.^2^ If applied within a limited time window, only the application of tissue plasminogen activator and mechanical thrombectomy serve as the gold standard of treatment.^3,4^ Adjuvant neuroprotection therapies are needed to improve survival in the hyper acute phase, reduce tissue damage in the subacute phase and thus improve long-term functional recovery and rehabilitation.

Ambroxol hydrochloride (2-amino-3,5-dibromo-*N*-methylbenzylamine hydrochloride) is a routinely used over-the-counter mucolytic drug that has recently gained attention as a possible neuroprotective agent.^5,6^ Microdialysis and ultra-high performance liquid chromatography coupled with mass spectrometry found that ambroxol easily penetrates the striatum of rats even at low dosages.^7^ Studies in mice and humans have also found therapeutic ambroxol concentrations in the brain.^8,9^ These properties, together with its low-toxicity profile, make ambroxol an optimal candidate for drug-repurposing.^10,11^ Currently, ambroxol is also being investigated in other neurodegenerative disorders such as Parkinson’s disease, amyotrophic lateral sclerosis and Gaucher’s disease.^8,12–14^ In these diseases, a major focus has been placed on ambroxol’s glucocerebrosidase-stimulating activity. For example, an *in vivo* study in a rodent model for Parkinson’s disease, showed that ambroxol has neurorestorative potential ^9^ attributed to glucocerebrosidase-stimulating activity. The glucocerebrosidase enzyme gained attention in this research field since point mutations of the *gba1* gene, which codes for this enzyme, represent a risk factor for developing Parkinson’s disease. In fact, ambroxol is already being tested as a novel disease-modifying treatment for Parkinson’s disease in several clinical trials^8,15^ following evidence that it leads to α-synuclein clearance. Moreover, in Parkinson’s disease, ambroxol has been shown to restore the activity of tyrosine hydroxylase and the dopamine transporter.^16,17^ Tyrosine hydroxylase uses O_2_ as a cofactor to catalyze the conversion of L-tyrosine to levodopa. L-tyrosine to levodopa in turn, is a precursor of the potent vasoactive neurotransmitters: norepinephrine and epinephrine. In summary, mechanisms by which ambroxol mediates neurorestorative effects in other neurodegenerative diseases could also play a role in neuroprotection during ischaemia.

Besides the aforementioned mechanisms, ambroxol has a potent blocking effect on Na^+^ channels and effects in Ca^2+^ homeostasis.^18,19^ We hypothesize that ambroxol may therefore hinder massive electrochemical shifts between neurons, glia cells and the interstitial space leading to reduction of oedema and excitotoxicity. Potentially, effects on the synthesis of vasoactive neurotransmitters through tyrosine hydroxylase activity by ambroxol, may also play a role in compensating blood flow and neuronal activity. Moreover, ambroxol has scavenging effects on reactive oxygen species (ROS) as well as anti-inflammatory effects.^6,10,20,21^ ROS causes oxidative stress and neuroinflammation, which subsequently leads to DNA damage and cell death.^22^ These specific effects could lead ambroxol to prevent further tissue injury and alter the pathophysiological cascade of events that result in apoptosis due to ischaemia. Specifically, we hypothesize that the inhibition of ionic transport mechanisms, leads to a reduction of basal energy consumption in the brain during therapy. A direct effect of this, would be a reduction of neuronal activity ultimately resulting in reduced oedema, reduced tissue injury by ROS and reduced cell death. This in turn could translate into improved disease survival, morbidity, and rehabilitation in patients.

*In vivo* MRI has proven to be a robust non-invasive tool^22–26^ to assess stroke volume, cytotoxic oedema, vasogenic oedema, necrosis, functional and structural connectivity in the brain.^27,28^ More precisely, MRI sequences, such as T2-weighted (T2W) images and diffusion-weighted images (DWIs), are the gold standard for the identification and characterization of stroke lesions.^29–34^ Structural connectivity can be evaluated using fractional anisotropy (FA) maps, calculated from multidirectional DWI, and provide consistent measurements of white matter integrity.^35,36^ Furthermore, resting state functional MRI (rs-fMRI) reveals information about the functional brain connectivity since it measures haemodynamic changes caused by neuronal activity as measured by the blood-oxygen-level-dependent (BOLD) response through the magnetic properties of oxyhaemoglobin and deoxyhaemoglobin.^37,38^ However, although these mentioned tools provide macroscopic and clinically-available measurements of function, they are limited in assessing metabolic changes on the cellular level, which is crucial to understand the molecular mechanisms and suggested therapeutics. *Ex vivo* nuclear magnetic resonance (NMR) spectroscopy allows the dissection of metabolic profiles that can be useful to understand alterations in stroke pathogenesis or as a result of treatment. In the presence of a therapy, variations in specific metabolite concentrations may indicate gross changes in cellular priorities.^39,40^

In this work, we apply a complex multimodality approach to thoroughly characterize and delineate the effects of ambroxol administration after IS induction in rats. Our evaluation provides a unique longitudinal study of IS, including evaluations of vasogenic oedema, cytotoxic oedema, stroke volume, functional connectivity, structural connectivity, behavioural tests, histology, and NMR-based tissue metabolomics.

## Materials and methods

Rats were evaluated longitudinally during and after IS induction using a 60-minute middle cerebral artery occlusion followed by reperfusion, as previously described.^41^ Exact surgical details are described in the Supplementary material. The longitudinal cohort consisted of MRI acquisitions, behavioural tests, metabolomics and *ex vivo* histology, under treatment with ambroxol or two different vehicle control solutions. As later described, following rigorous statistical testing, the two different control groups are combined to make one single control group against ambroxol.

### Experimental animals

For the experimental setup, following ethical guidelines, an analysis plan was drawn and evaluated by local veterinarian advisors of the University of Tuebingen. Formal sample size and power calculations were conducted, moreover the experimental setup was approved by the local authorities (Regierungspraesidium Tuebingen). Healthy male Sprague Dawley rats (*n* = 53, Charles Rivers Laboratories, Germany) were housed in groups in standard individual ventilated type IV Makrolon cages (IVC, Tecniplast, Hohenpeißenberg, Germany). Animals were fed with standard food pellets and had unlimited access to water (further details in Supplementary material).

### Treatment

Treatment was administered intraperitoneally twice a day, starting directly after surgery ∼10 min after reperfusion (∼70 min after stroke onset) for 1 week. Ambroxol was administered at a dosage of 90 mg/kg daily in two equally divided doses diluted in an equal part solution of 0.9% sodium chloride (NaCl) and polyethylene glycol 400 (PEG; Sigma-Aldrich, St. Louis, Missouri, USA). Following positive stroke lesion induction during occlusion using diffusion MRI, animals received either ambroxol (*n* = 17), NaCl solution (*n* = 17) or the vehicle solution PEG/NaCl (PEG, *n* = 19). No statistically pre-planned randomization or blinding scheme was used for group selection or therapy administration. Animals were excluded from analysis (*n* = 20) if the middle cerebral artery occlusion surgery was not successful and if the animals died peri- or post-surgery without post-therapy imaging. Complete details of animals excluded as well as animal numbers per experiment are described in Supplementary materials and detailed in Supplementary Tables 1 and 2.

### Experimental setup

Baseline MRI scans were performed before surgery in order to discard anatomical abnormalities and establish baseline reference values. A stroke area can be detected in the hyper-acute stroke phase by evaluating the apparent diffusion coefficient (ADC) of the brain tissue, which is calculated from DWI.^42^ Therefore, a DWI was performed (occlusion MRI) while the nylon filament was occluding the MCA, approximately 40–55 minutes post-filament insertion. Follow-up MRI measurements and behavioural tests were performed at 24 h, 72 h, 1 week, and 1 month post-stroke surgery. The follow-up imaging protocol contained T2W and multidirectional-DWI sequences for all time points. ADC maps and FA maps were calculated from DWI. ADC in the stroke region was evaluated because it is a biomarker for water diffusion restriction due to cellular swelling.^43–45^ In fact, ADC lesions are highly correlated to final stroke lesions in histology.^34,41^ T2W images, on the other hand, allow the evaluation of vasogenic oedema, which provides a measurement of water accumulation in stroke tissue due to a mixture of blood-brain barrier breakage and tissue necrosis.^30,46^ rs-fMRI sequences were obtained at baseline, 24 h, and 1 month. The imaging data were pre-processed and analyzed as previously described by Ionescu *et al.*^47^ Subsets of animals were sacrificed at every imaging time point and excised brains were preserved either for histology or metabolomic analyses. Precise details on MRI acquisition and post-processing are provided in the Supplementary materials.

### Volumes of interest

Stroke region volumetry was performed using MRI. During occlusion stroke volumetry was evaluated using ADC images, since T2-lesions are not present in the hyper-acute stroke phase. The stroke lesions during occlusion (before therapy start) are used as start-off stroke lesions and are calculated by applying an individually defined threshold of 22% below the baseline median for each animal on the ipsilesional hemisphere, as previously described for hyper-acute stroke lesion segmentations.^48^ We have shown before that manual delineation of the whole stroke region volume at 24 h using volumes of interest (VOI) that combine the use of ADC and T2W images for delineation correlates strongly to end-point histological segmentations.^41^ Therefore, for the subsequent time points after occlusion, VOIs of the complete stroke regions were delineated by two blinded preclinical neuroimaging investigators in the entire 3D brain volume at an image resolution of 200 µm^3^ using MATLAB (V. R2019b, The MathWorks, Inc., Natick, USA) as previously performed.^41^ Volumes for the striatum and adjacent cortical stroke lesions were also segmented in their entirety over the whole brain. Contralateral template regions such as the striatum and cortex were imported from the Schiffer brain atlas.^49^ For FA images, VOIs were drawn by hand around the corpus callosum using the whole brain following anatomy in the Paxinos brain atlas^50^ and divided into internal capsule, external capsule, and genu. Resting-state functional connectivity (rs-FC) analysis was performed using the 52 regions selected from the Schiffer rat brain atlas in the Paxinos space (Supplementary Table 7).^49,50^

### Behavioural experiments

Brain function can be evaluated using behavioural tests to determine functional deficits after stroke.^51^ All animals received a baseline behaviour evaluation after one week of habituation to the facility. We then performed behavioural evaluations, including the beam walk test, sticky label test, and grip test, to provide a longitudinal measure of functional brain outcome throughout the experiment (exact details of experimental setup in Supplementary material and Supplementary Table 2). Data are presented as mean and standard deviation.

### *Ex vivo* NMR tissue metabolomics

Brain samples from a randomly selected subset of nine rats from the imaging cohort at 24 h (ambroxol *n* = 4, control *n* = 5) were quenched in liquid nitrogen directly after dissection and sectioned at 20°C into ipsi- and contralesional hemisphere. To account for lesion heterogeneity and stroke severity, the striatum and cortex were excised into portions. The striatum was excised into three equal parts spanning from the base of the brain towards the cortex. The cortex was divided into two samples, one medial and one lateral. Storage occurred at −80°C until analysis. All samples were cryogenically pulverized (Covaris CryoPrep^™^ CP02, Woburn, MA, USA) and prepared by polar metabolite extraction routines as previously described.^52^^1^H NMR spectra were recorded by a 14.10 Tesla (600 MHz for proton channel) ultra-shielded NMR spectrometer with a 1.7 mm room temperature microprobe (Avance^™^ III HD, Bruker BioSpin, Karlsruhe, Germany). Carr-Purcell-Meiboom-GillI experiments were used for metabolites annotation and quantification, employing commercial software (ChenomX NMR Suite 8.5) and integrated databases (ChenomX and HMDB libraries). Specific details are described in supplementary materials.

### Histology

Randomly selected animals from both groups at 1 month were used for haematoxylin and eosin. Haematoxylin and eosin was performed on two to six slides for each animal (*n* = 5 NaCl, *n* = 2 PEG for controls and *n* = 4 for ambroxol). Immunohistochemistry staining was used to demonstrate activated glia cells and apoptosis around the stroke area using glial fibrillary acidic protein and Caspase 3 as previously described.^41^ Details in Supplementary materials.

### Statistical analysis

One-way ANOVA was used to evaluate the initial pre-therapy stroke volume of the three experimental groups. This analysis was used to confirm adequate stroke volume distributions between the groups previous to therapy administration. Repeated measures ANOVA (rm-ANOVA) was then used to evaluate main group, time, and interaction of group and time between the two vehicle control groups. This was performed to discard therapy effects produced by PEG. Since there were no differences between the two control groups (Supplementary Table 3), we consolidated them into a single control group.

We evaluated the ambroxol and the combined control group using rm-ANOVA. Longitudinal imaging and behavioural data were evaluated for main group, time, and interaction of group and time effects. Fisher’s least significant difference post-hoc test was used for multiple comparison and to point out specific statistical significances following rm-ANOVA. Results of the rm-ANOVA are shown with *P* values in the main manuscript together with an index directing to the corresponding degrees of freedom and F-tests (F) in Supplementary Tables 3 and 4.

NMR tissue metabolomics statistical analysis was performed with the MetaboAnalyst 5.0 web server.^53^ Striatal and cortical metabolite data were analyzed using unpaired *t*-tests comparing control against ambroxol groups for every metabolite. Due to the high number of comparisons for metabolomics, as well as for in rs-FC analysis, here multiple comparisons were corrected with a specific false discovery rate correction (FDR correction) according to Benjamini-Hochberg.^54^ In addition, two-tail student’s *t*-tests were performed to point out relevant and specific comparisons as specified in the text. Chi² tests were used for the evaluation of the success rate for the beam walk and sticky label test since they consist of dichotomic variables.

An alpha level of 0.05 and a 0.95 confidence interval was used for all applicable statistical evaluations. Boxplots show the median value and the 25–75th percentiles. The whiskers represent the minimum and maximum values of the datasets. If not otherwise stated, results are presented as median and range (min–max). All statistical analyses except NMR were performed using MATLAB.

## Results

All animal numbers for imaging and behavioural experiments are listed for the different time points in Supplementary Tables 1 and 2. Full details of the parameters for statistical analysis as well as ANOVA are indexed per result to their corresponding values in Supplementary Tables 3 and 4.

### Stroke lesion volume during occlusion, pre-therapy

Results from stroke volumetry using MRI during occlusion showed that the groups were statistically similar, previous to administration of therapy. Specifically, one-way ANOVA was used to evaluate the stroke volumes of the ambroxol, PEG, and NaCl groups during occlusion, with no significant differences (*P* = 0.21). This result confirmed that the distribution of animals into the three distinct groups did not have initial biases in stroke volume.

In order to exclude any possible effects of the vehicle component PEG in our experiments, we evaluated it directly to the NaCl control group. We investigated if the MRI stroke volumes, ADC and T2 of the stroke regions, white matter integrity or behaviour were statistically different between the two control groups using rm-ANOVA (Supplementary Table 3 index no. 1–5). From all tests, 15 out of 16 evaluations yielded no significant results [range of *P*-values = (0.34–0.99)]. Only the grip test showed a singular significant main group difference (*P* = 0.02), which was no longer significant after multiple comparison correction. Due to the overwhelming multimodal similarity between the NaCl and PEG groups, the groups were pooled together as a single-vehicle control group to be evaluated against the ambroxol group.

### Ambroxol reduces stroke volume in the acute stroke phase

**Figure 1:**
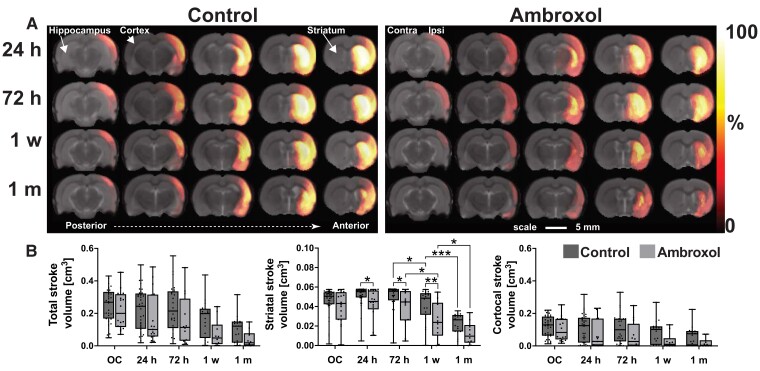
**Longitudinal stroke region development over time.** (**A)** A stroke lesion mask (or volume of interest) was produced manually for every animal at every time point. These masks were voxel-wise averaged and are presented as a heatmap overlay on the anatomical brain images. Maximum intensity of this heat map overlay represents 100% of all animals presented a stroke in that specific voxel. The watershed heatmap intensity bar reflects the percentage frequency of a stroke lesion per voxel. **(B)** Total stroke, total striatal and total cortical stroke volumes were quantified. Each boxplot shows the median value (central line), individual animals (data points) and the 25th to 75th percentiles. Whiskers represent the minimum and maximum values. rm-ANOVA was performed for statistical analysis, followed by Fisher’s test. rm-ANOVA results are presented in Supplementary Table 4 index no. 1–3. * refers to *P* < 0.05, ** refers to *P* < 0.01 and *** refers to *P* < 0.001. OC, occlusion.

In general, animals in the ambroxol and control groups presented highly variable stroke volumes located either solely in the striatum or in both striatum and cortex in the entire cerebrum. For better representation of the stroke volume variability, we produced an average image of all the stroke volumes per group and time at different anatomical levels (Fig. 1A). This allows the visualization of the extent of the stroke lesion in multiple brain areas. Stroke volume quantifications are shown in Fig. 1B. rm-ANOVA found a main group effect between control and ambroxol in the whole stroke region (*P* < 0.001, Supplementary Table 4 index no. 1) and in the striatum (*P* < 0.001; Supplementary Table 4 index no. 2). Post-hoc testing pointed to stroke volume reductions in the striatum of ambroxol treated animals at 24 h (*P* = 0.02), 72 h (*P* = 0.02) and 1 week (*P* = 0.003) after stroke induction. rm-ANOVA revealed a significant group difference in the cortical stroke region (*P* < 0.001, Supplementary Table 4 index no. 3), which is also observable in the cortical regions surrounding the hippocampus in the ambroxol animals (Fig. 1A), but post-hoc testing found no differences between the time points. In general, the stroke volumes of both ambroxol and control significantly reduced over time (*P* < 0.01). There were no significant volume differences between the groups at 1 month. In summary, these experiments showed that in comparison to controls, there was a statistically higher reduction of stroke volume in rats injected with ambroxol, in the striatal region starting at 24 h post-therapy with a maximum difference at 1 week. Moreover, although stroke volume reductions in the cortex of ambroxol animals are also clearly noticeable and are recognized by rm-ANOVA, post-hoc testing did not point out any differences.

### Vasogenic oedema reductions under ambroxol therapy

**Figure 2:**
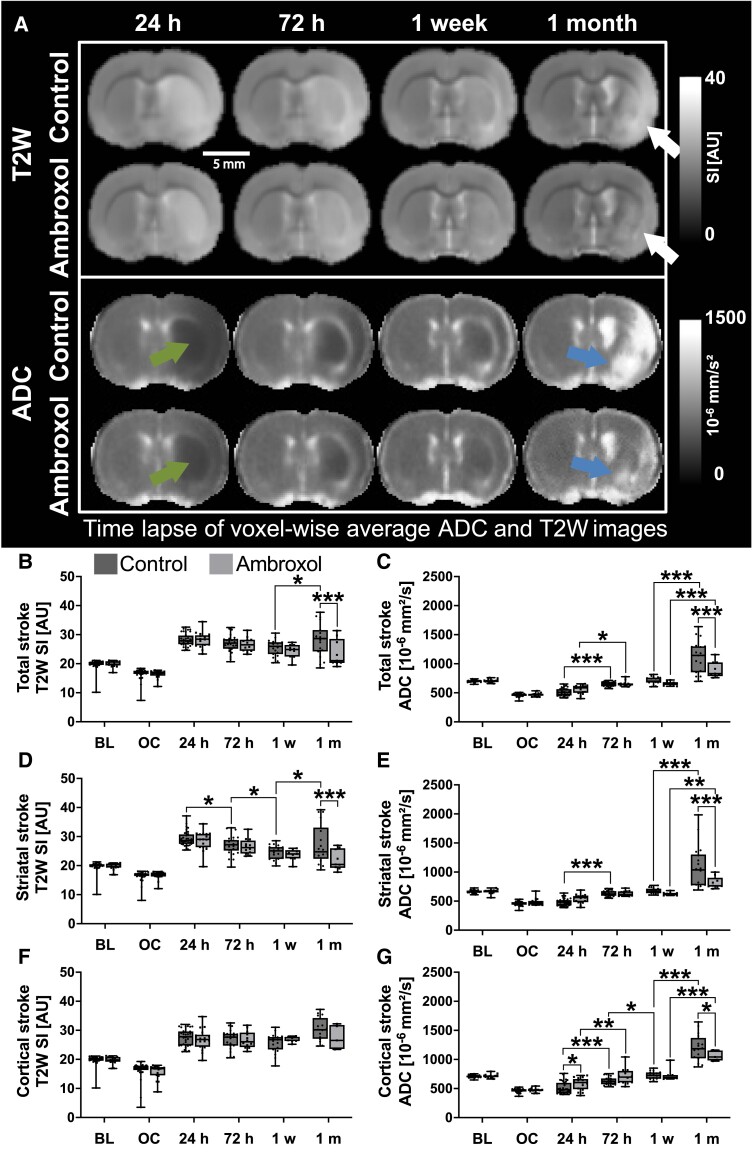
**T2W signal intensity and apparent diffusion coefficient (ADC).** (**A)** coronal examples of voxel-wise average T2W and ADC images of all rats are shown overtime. The arrows at 24 h point areas of reduced ADC preceding the areas of necrosis indicated by the arrows at 1 month in the T2W and ADC images in both animal groups. This effect is more predominant in controls than ambroxol animals. T2W and ADC quantifications are shown for the total stroke volume, striatum and cortex **(B–G).** Control animals presented significantly higher T2 signal intensity at 1 month in total stroke and striatum. Cytotoxic oedema was most intense at 24 h, preceding significant increments at 1 month. Each boxplot shows the median value (central line) and the 25–75th percentiles. rm-ANOVA was performed for statistical analysis, followed by Fisher’s multiple comparison test. rm-ANOVA results are presented in Supplementary Table 4 index no. 4–6 (T2W) and no. 10–12 (ADC). Whiskers represent the minimum and maximum values. * refers to *P* < 0.05, ** refers to *P* < 0.01 and *** refers to *P* < 0.001. BL, baseline; OC, occlusion.

We evaluated the T2W signal intensity over time, since it is an imaging marker of vasogenic oedema (Fig. 2). rm-ANOVA revealed that vasogenic oedema in the total stroke region was significantly different between the ambroxol and control group (*P* = 0.014, Supplementary Table 4 index no. 4). There was also a significant interaction between timepoint and animal group (*P* = 0.046). Post-hoc analysis showed a significant difference between the ambroxol and control group at 1 month (*P* < 0.001; Fig. 2A, white arrow). Additionally, the total stroke volume of control animals presented significantly higher increments in T2W signal intensity from the 1 week to the 1-month time point in comparison to ambroxol (*P* = 0.014). rm-ANOVA showed that the striatal stroke T2W signal was significantly different between the groups (*P* = 0.01; Fig. 2D and Supplementary Table 4 index no. 5). It also showed a significant interaction between group and time (*P* = 0.034). Here, post-hoc striatal analysis showed again a significant increment on T2W signal intensity at 1 month for the control group (*P* < 0.001). Moreover, the control group also showed significant dynamic changes of T2W signal intensity between different time points, especially from 1 week to 1 month (*P* = 0.02). On the contrary, T2W signal intensity of ambroxol treated animals returned to values close to baseline after 1 month. These results were further confirmed by normalizing the T2W signal intensity to the contralateral hemisphere, where a similar significant increment in T2W signal intensity is observed in controls (Supplementary Fig. 1A).

rm-ANOVA found no significant differences between groups in the cortical brain regions (Figs. 2F and 1A; Supplementary Table 4 index no. 6). Altogether these results demonstrate that the control animals presented significantly higher vasogenic oedema than ambroxol animals in the acute phase. Moreover, T2W signal was significantly reduced in the stroke lesions of ambroxol animals at one month in comparison to controls, which reflects a reduction of liquid necrotic regions.

### Stroke lesion severity is ameliorated under ambroxol therapy

Strong reductions of ADC in the acute stroke phase represent cytotoxic oedema, which is commonly used as a biomarker of stroke severity. We show the average ADC images of all evaluated brains and the results of the quantification in Fig. 2A. rm-ANOVA revealed a main group difference in the whole stroke (*P* = 0.023, Supplementary Table 4 index no. 10) and in the striatal stroke (*P* = 0.02, Supplementary Table 4 index no. 11) as well as interactions with time (*P* < 0.001). Following one day of therapy, ADC in the ambroxol group was slightly more reduced than in control animals in the whole stroke volume. Here, a student *t*-test revealed an initial significant ADC reduction (*P* = 0.02 in Fig. 2C; green arrow, Fig. 2A); however, no significance was found in the post-hoc test. At 1 month however, there were significant increments of ADC in controls over ambroxol (*P* < 0.001), consistent with the T2W signal increments. The situation was similar in the striatum (Fig. 2E) where t-testing also showed an initial reduction of ADC in controls at 24 h in the striatum (*P* = 0.01), yet not in post-hoc testing. Multiple comparison correction again revealed significant increments in ADC of control animals at 1 month in comparison to ambroxol (*P* < 0.001). Increased ADC as shown in Fig. 2A (blue arrow) is consistent with necrotic liquefaction of the brain tissue, which was confirmed in histology (Supplementary Fig. 2). rm-ANOVA showed a significant interaction between time and group in the cortex (*P* < 0.02), an effect of time (*P* < 0.001) but no main group effect (*P* = 0.8, Supplementary Table 4 index no. 12). Here, post-hoc testing showed that ambroxol animals presented less severe ADC in the cortex at 24 h (*P* = 0.05) than control animals, which is clearly visible in the voxel-wise images (Fig. 2A, 24 h). This finding aligned well, with our observed reductions of stroke volume in the cortex. At 1 month, ambroxol animals also presented less increments of ADC consistent with liquid necrosis (*P* = 0.03) as seen in Fig. 2A and G. These results are further supported in the normalized ipsilateral/contralateral ratio analysis (Supplementary Fig. 1B). Altogether, our data points out that ambroxol-treated animals presented less cytotoxic oedema at 24 h followed by subsequent significantly higher increments in water fluidity both in the striatum and in the cortex at 1 month (Fig. 2A, arrows). The results perfectly match the increased T2W signal intensity at 1 month, demonstrating water and diffusion values compatible with necrotic liquefaction of brain tissue confirmed in histology.

### Improved behavioural outcomes in ambroxol-treated animals

**Figure 3:**
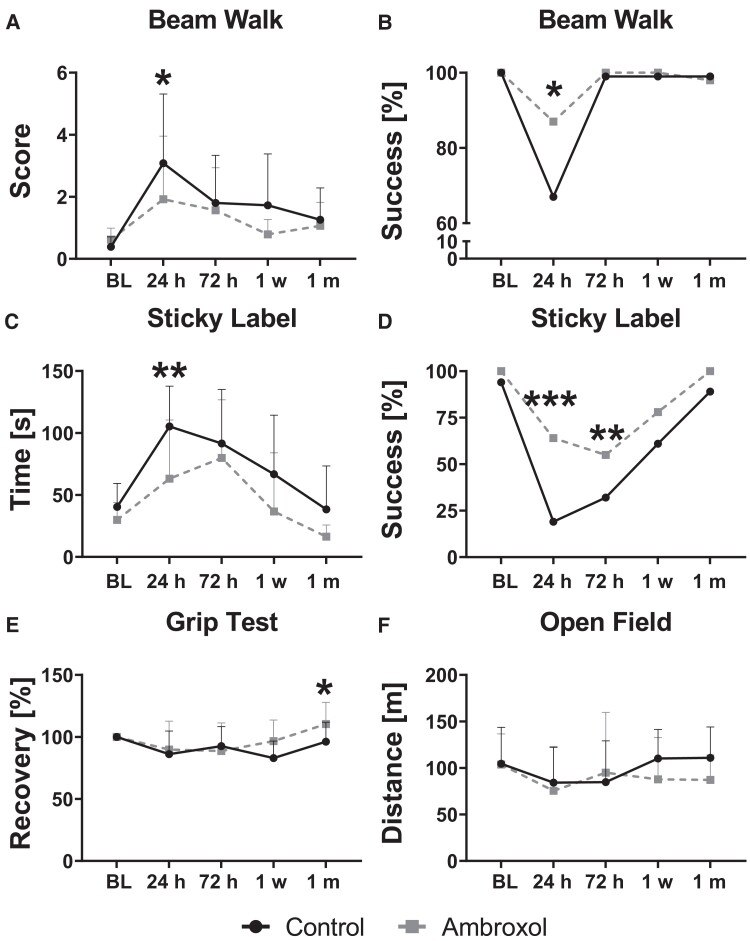
**Behavioural outcome of rats before and after stroke surgery.** (**A)** Balance and coordination were measured in animals by crossing a narrow beam and evaluated using a score system. **(B)** Actual success of crossing the narrow beam was tabulated. **(C)** The time required for an animal to remove a sticky piece of tape from the forepaws was measured to evaluate sensory and motor deficits longitudinally. **(D)** The sticky label success rate was also tabulated. **(E)** The recovery rate of strength in the forepaws was used to evaluate recovering motor deficits and calculated using the baseline value as 100%. **(F)** Travelled distance in an open field was measured to evaluate anxiety and exploratory behaviour. All data points represent the mean value and the error bars represent the SD in one direction. rm-ANOVA was performed for statistical analysis, followed by Fisher’s test for the graphs A, C, E, and F. Chi^2^ test was performed for statistical analysis for data in graphs B and D. rm-ANOVA results are presented in Supplementary Table 4 index no. 16–19. * refers to *P* < 0.05; ** refers to *P* < 0.01; *** refers to *P* < 0.001.BL, baseline.

We evaluated behaviour using tests that focused on sensory, motor and coordination tasks at every imaging time point (Fig. 3). Specific details of the tests are provided in Supplementary material. The beam walk test focuses on combined sensory-motor skills and coordination. We scored the results for every animal and evaluated them using rm-ANOVA, which showed a significant main group effect (*P* = 0.036, Supplementary Table 4 index no. 16). Multiple comparison results revealed that at 24 h, ambroxol animals performed significantly better than control animals (*P* = 0.03, Fig. 3A). Since stroke deteriorated the mood and compliance of the animals to perform tasks, we also evaluated the beam walk test as a dichotomic variable (completed or not completed test) using Chi² test statistics. The results confirmed that ambroxol treated animals were more successful than controls at crossing the beam walk at 24 h (*P* = 0.02, Fig. 3B). Overall, the strongest effect of ambroxol on the beam walk test was observed at 24 h.

The sticky label test provides information on sensory deficits produced by the stroke lesion. Here rm-ANOVA also showed a significant main group effect (*P* < 0.001, Supplementary Table 4 index no. 17). Specifically, this effect was significant at 24 h, showing a worse sensory deficit in control animals (*P* = 0.001, Fig. 3C). Success to finish the sticky tape removal was also tabulated and analyzed using the Chi² test yielding a significant deficit for controls at 24 and 72 h (*P* < 0.001 and *P* < 0.01 respectively, Fig. 3D). Altogether, the sticky label test revealed stronger deficits in controls animals than ambroxol treated animals at 24 and 72 h.

The grip test was used to evaluate the motor function of the animals following stroke. rm-ANOVA found a significant main effect (*P* = 0.035; Supplementary Table 4 index no. 18). Interestingly, motor function was significantly improved in ambroxol animals at 1 month in comparison to controls (*P* = 0.04, Fig. 3E). Finally, we performed an assessment of anxiety and exploration using the open field test. This test did not show significant differences in rm-ANOVA with a main group effect: *P* = 0.201 and an interaction of *P* = 0.47 (Fig. 3F and Supplementary Table 4 index no. 19).

Altogether the behavioural experiments determined that there was a significant improvement in coordination and sensory tasks at 24 and 72 h, while motor skills were improved at 1 month after stroke onset under ambroxol therapy.

### Structural connectivity changes of the corpus callosum

**Figure 4:**
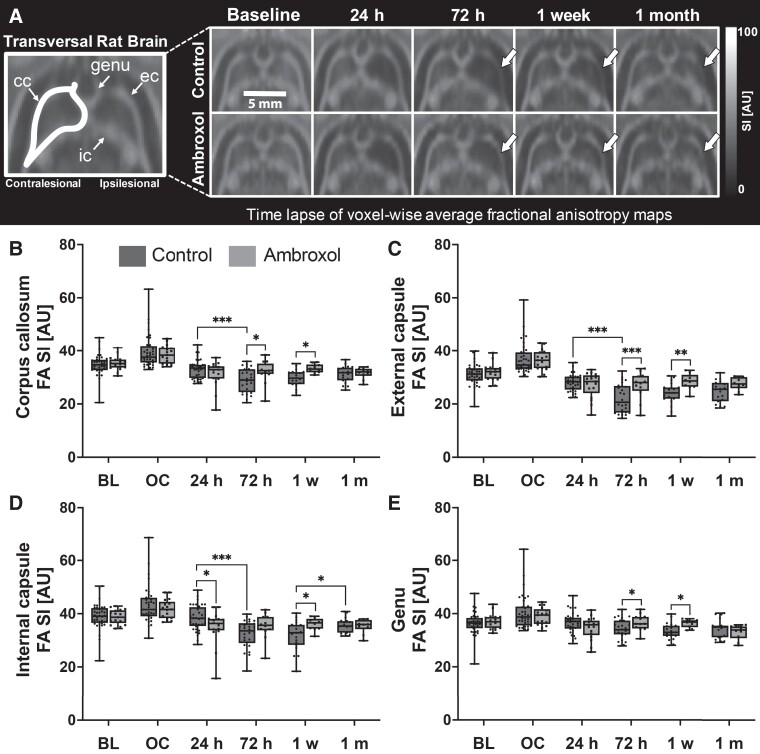
**Longitudinal white matter degeneration in the corpus callosum.** (**A)** Voxel-wise average FA images of all evaluated animals are shown over time for both groups. Quantification of FA data extracted from the corpus callosum **(B)** and the external capsule **(C)** is shown in the graphs. The external capsule presented significant white matter degeneration at 72 h and 1 week (arrows). The internal capsule **(D)** and the genu **(E)** of both groups presented a small decline in FA starting at 24 h with slightly better temporal recovery in ambroxol animals than controls. Each boxplot shows the median value (central line) and the 25th to 75th percentiles. Whiskers represent the minimum and maximum values. rm-ANOVA was performed for statistical analysis, followed by Fisher’s test. rm-ANOVA results are presented in Supplementary Table 4 index no. 20–23. *Refers to *P* < 0.05 and ** refers to *P* < 0.01. CC, corpus callosum; EC, external capsule; IC, internal capsule; BL, baseline; OC, occlusion.

FA maps calculated from DWI provide a measure of white matter integrity through the estimation of directional diffusivity produced by neuronal axons. We evaluated FA over time to determine the degree of white matter degeneration following IS. Figure 4A shows voxel-wise-average FA images of all the coregistered animals per group and time point. rm-ANOVA showed a significant group difference in the whole corpus callosum (*P* = 0.042) as well as a time interaction (*P* = 0.006, Supplementary Table 4 index no. 20). Here, at 72 h and at 1-week post stroke, post-hoc analysis showed significant differences between the groups (*P* < 0.05, Fig. 4B). Additionally, we used rm-ANOVA to separately evaluate the external capsule, internal capsule and the genu (Fig. 4C–E). The external capsule presented main group differences using rm-ANOVA (*P* < 0.001, Supplementary Table 4 index no. 22) as well as a significant time-group interaction (*P* = 0.007) The internal capsule and genu showed significant interactions of time and group (*P* = 0.004 and *P* = 0.006, Supplementary Table 4 index no. 21 and 23). There was significantly increased structural degeneration in the external capsule of control animals at 72 h and 1 week, in contrast to ambroxol animals (Fig. 4C, 72 h: *P* < 0.001, 1 week: *P* = 0.006). Interestingly, the internal capsule showed statistically lower FA for the ambroxol at 24 h than for control animals (*P* < 0.05). However, in the internal capsule, the FA of control animals continues to decline over time, while the FA of ambroxol animals recovers and remains relatively constant during therapy, producing a difference between the groups at 1 week (*P* < 0.05). The genu presented significant changes between the groups at 72 h and 1 week (*P* < 0.05 on both time points). Altogether the VOI analysis demonstrates relevant significant changes in FA of the different parts of the corpus callosum over time, demonstrating the dynamics of white matter damage in stroke lesions. Moreover, we showed that ambroxol animals presented an ameliorated white matter damage than control animals during therapy administration.

We were interested in the visualizing the most relevant changes in white matter integrity. Therefore, we additionally calculated voxel-wise *t*-test images between control and ambroxol for every single time point. This data evaluated the exact location of white matter differences between the datasets and can be found in Supplementary material (Supplementary Fig. 3).

### Functional connectivity

rs-fMRI consists of the measurement of rs-FC by computing interregional correlations of the BOLD signal between different brain regions.^55^ In fact, the BOLD fluctuations (or rs-FC) have been strongly correlated to structural neuroanatomical brain networks.^56,57^ To further understand the relationship between behavioural deficits and structural brain connectivity, we evaluated brain function non-invasively using *in vivo* rs-fMRI. First, we calculated rs-FC on a whole brain level, which showed more significant correlations on the control animals than on ambroxol at 24 h (Supplementary Figs. 4 and 5). In comparison to baseline, global Pearson’s r reduced for both groups from *r* = 0.42 (baseline control) and *r* = 0.39 (baseline ambroxol) to *r* = 0.36 and 0.25 under therapy at 24 h, correspondingly. At 1 month, the median Pearson’s *r* for ambroxol animals reached *r* = 0.32, while connectivity for control animals remained at *r* = 0.34. These findings show that under ambroxol administration at 24 h, there is a global reduction in functional brain connectivity, in comparison to the similar global brain connectivity at baseline and at 1 month.

**Figure 5:**
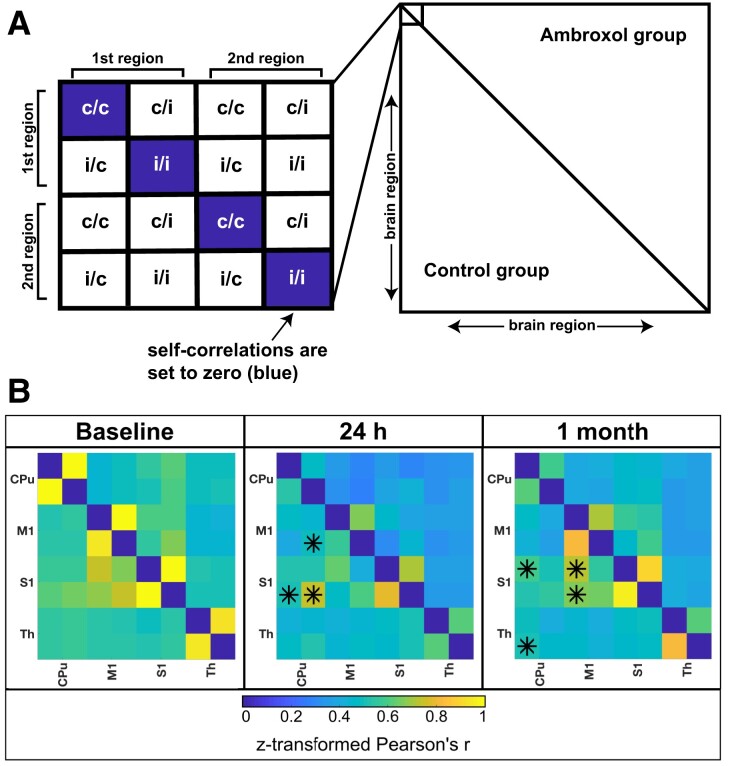
**Resting-state functional connectivity between the groups in motor pathway.** Pair-wise rs-FC of regions belonging to the motor network are shown as a heat map with strongest correlations as bright and weakest as dark (z-transformed Pearson’s *r*). **(A)** A schematic shows an explanation for readability of the correlation map. The y-axes and the x-axes show the correlated brain regions. The lower triangle represents the connectivity of the control group and the upper triangle represents the connectivity of the ambroxol group. For each brain region the contralesional hemisphere (c) is presented first in y and x direction, the ipsilesional hemisphere (**i**) second. Self-correlations were set to 0 (dark diagnonal line). **(B)** Connectivity maps between the groups at baseline, 24 h and 1 month post stroke induction for the motor pathway are shown. Control group: *n* = 17; ambroxol group: *n* = 9. rm-ANOVA was performed for statistical analysis, followed by FDR correction. Significances found by the rm-ANOVA are marked with an asterisk, referring to a statistically significant higher connectivity in the respective group at *P* < 0.05. CPu, caudate putamen; M1, primary motor region; S1, somatosensory region; Th, thalamus; BL, baseline.

**Figure 6:**
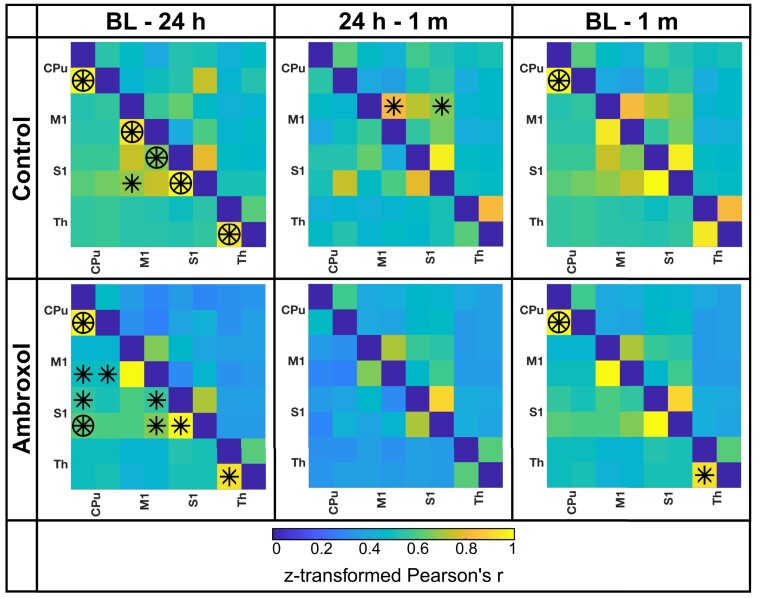
**Resting-state functional connectivity of motor pathway compared for both groups between time points.** The diagonal dark line through the middle of each graph shows self-correlations which are set to zero. The lower left triangle represents the connectivity of the first time point (titled first) and the upper right triangle represents the second time point (titled second). Connectivity maps comparing the time-points baseline to 24 h, 24 h to 1 month and baseline to 1 month are shown. Control group: *n* = 17; ambroxol group: *n* = 9. Different time points were analyzed using rm-ANOVA followed by FDR correction. Significances found are marked with an asterisk referring to a significant higher connectivity with a *P*-value < 0.05 for the respective time point before FDR correction. Circled asterisks remained significant after FDR correction. CPu, caudate putamen; M1, primary motor region; S1, somatosensory region; Th, thalamus; BL, baseline.

Then, we focused specifically on the motor network (Figs. 5 and 6). We found a clear reduction of rs-FC in different brain regions of the motor network in both groups at 24 h (Fig. 5), but even lower rs-FC in the ambroxol treated animals (control group, Pearson’s *r* = 0.49, ambroxol, Pearson’s *r* = 0.36). Without FDR correction at 24 h post-stroke, control animals presented significantly higher rs-FC between the striatum (CPu) of both hemispheres and the ipsilesional somatosensory cortex (S1) than ambroxol-treated animals. Interestingly, there was also increased connectivity at 24 h between the ipsilesional striatum and the ipsilesional motor cortex (M1) in the control group animals. In summary, the motor network of ambroxol-treated animals did not increase network rs-FC at 24 h post-stroke, consistent with the global connectivity result. Moreover, the control animals present increased functional connectivity of the sensory motor cortex at 24 h in comparison to ambroxol animals.

At 1 month post stroke induction (Fig. 5B), control animals presented a significantly higher rs-FC between the contralesional striatum and the contralesional S1 compared to ambroxol animals. There was also a significant increment in rs-FC of control animals between the contralesional striatum and the ipsilesional thalamus compared to ambroxol animals. Moreover, in control animals, bilateral somatosensory brain regions presented significantly increased rs-FC to the contralesional M1 stroke cortex. This effect was not present in ambroxol animals at 1 month. Altogether, in the chronic stroke phase, control animals present increased connectivity patterns in the sensory motor cortex, which are not found in ambroxol treated animals.

We evaluated statistical differences in rs-FC of the motor network between different time points (Fig. 6 and Supplementary Fig. 5). Here, we confirmed reduced correlations in the motor pathway and reduced connectivity in ambroxol-treated animals between baseline and 24 h, in comparison to controls. FDR correction showed the most prominent reduced correlations in the control group. This finding is interesting because while there is a global connectivity reduction of the motor pathway of ambroxol-treated animals (Fig. 6, baseline—24 h), the connectivity of the motor pathway in control animals has more reduced rs-FC regions. This is congruent with the starker behavioural deficits at 24 h of control animals.

Analysis of the 24 h—1 month time points for ambroxol animals showed no rs-FC differences. However, global rs-FC of the sensorymotor network is more attenuated at 24 h (*r* = 0.25, during ambroxol therapy) than at 1 month (*r* = 0.32, 3 weeks after the last ambroxol dosage). In contrast, the control group presents an unchanged global rs-FC motor network connectivity between both time points (24 h: *r* = 0.36; 1 month: *r* = 0.34) with only a few increased rs-FC regions at 1 month without FDR correction.

The comparison of baseline to 1 month showed significantly reduced rs-FC at 1 month between the ipsi- and contralesional striatum for both animal groups with similar connectivity between groups and between time points (control group: 43% reduction, from *r* = 1.10 to *r* = 0.62 and ambroxol group: 44% reduction, from *r* = 1.04 to r = 0.59). Furthermore, we found a significantly reduced rs-FC for the ambroxol group between the ipsi- and contralesional thalamus (34%, reduction from *r* = 0.93 to *r* = 0.61). In summary, the time point analysis shows a total rs-FC reduction of the motor network at 24 h for ambroxol animals and only focal rs-FC reductions in control animals.

### Metabolomics

Changes in metabolic pathways can shed further light on future biomarkers or drug targets. These alterations can be helpful to understand the observed macroscopic effects and provide interpretability. We present results from 49 different metabolites in striatal and cortical samples from the ipsi- and contralesional hemisphere of a subset of stroke animals from the imaging cohort belonging to control (*n* = 5) and ambroxol groups (*n* = 4). For easiness, the most relevant metabolites are summarized in Table 1. The complete range of metabolites is shown in Supplementary Table 5. *t*-test statistics were performed to find significant differences between therapy groups in the striatum and cortex samples. FDR correction was also performed to correct for multiple comparisons. However, due to the high number of comparisons and reduced number of replicates, FDR correction dissolved all the meaningful and interesting significances found by *t*-testing between ambroxol and control. We present the *t*-test significances, since we consider that they have strong hypothesis-formulating value.

**Table 1:** Most relevant metabolites studied. Ambroxol and control metabolomic brain data in average fold changes (FC, log2 scale) in the contralesional and ipsilesional cortex and striatum

		Cortex				Striatum			
		Contralesional		Ipsilesional		Contralesional		Ipsilesional	
Description	Metabolite	[log_2_ FC]		[log_2_ FC]		[log_2_ FC]		[log_2_ FC]	
Energy metabolism	AMP	↑*	0.4	↓	0.2	↑	0.1	↓	0.4
	NAD	↑*	1.0	↑	0.3	↑	0.5	↑	0.3
	Phospho-creatine	↑	1.2	↑	1.2	↑	0.5	↑*	1.7
Vitamin	Pantothenate	↓	1.5	↑*	1.4	↑	0.3	↑*	1.0
Glycolysis	Glucose	↓	1.0	↑	1.0	↓*	1.1	↑	0.7
	Lactate	↑	0.1	↑	0.1	↓	0.1	↓*	0.3
Krebs cycle (TCA)	Citrate	↓	1.0	↑	0.1	↓*	1.1	↑	0.7
Short chain fatty acid	Acetate	↓*	1.1	≈	0.0	↓*	0.9	↓	0.3
Branched-chain amino acid	Valine	↑*	0.8	↑*	0.8	↑	0.2	≈	0.0
Amino acid metabolism	Aspartate	↑*	0.4	≈	0.0	≈	0.0	↓	0.2
	Lysine	↓	1.0	↑	0.3	↓*	1.1	↑	0.3
	Tyrosine	↑	0.7	↑*	1.3	↑	0.5	↑	0.5
Ketone bodies	Acetone	↓	0.2	↑*	1.7	↓*	1.1	↑	0.9
	3-Hydroxy-butyrate	↑	0.4	↑*	1.4	↑	0.1	↑	0.2
Cell membrane synthesis, Kennedy pathway	Acetylcholine	↑*	0.8	↓	0.7	↑	0.2	≈	0.0
	Choline	↓*	0.6	↑	0.7	↓	0.6	↑	0.1
	Ethanolamine	↑	0.9	↓	0.5	↓	0.1	↓*	1.1
Redox metabolism	Glutathione	↑*	0.9	↑	1.5	↓	0.6	↑*	1.3

AMP, adenosine monophosphate; NAD, nicotinamide adenine dinucleotide; Contr, contralesional; Ipsi, ipsilesional. *t*-test comparisons were made between the control and ambroxol groups.

* *P*-value < 0.05. The results are not significant after FDR.

**Figure 7:**
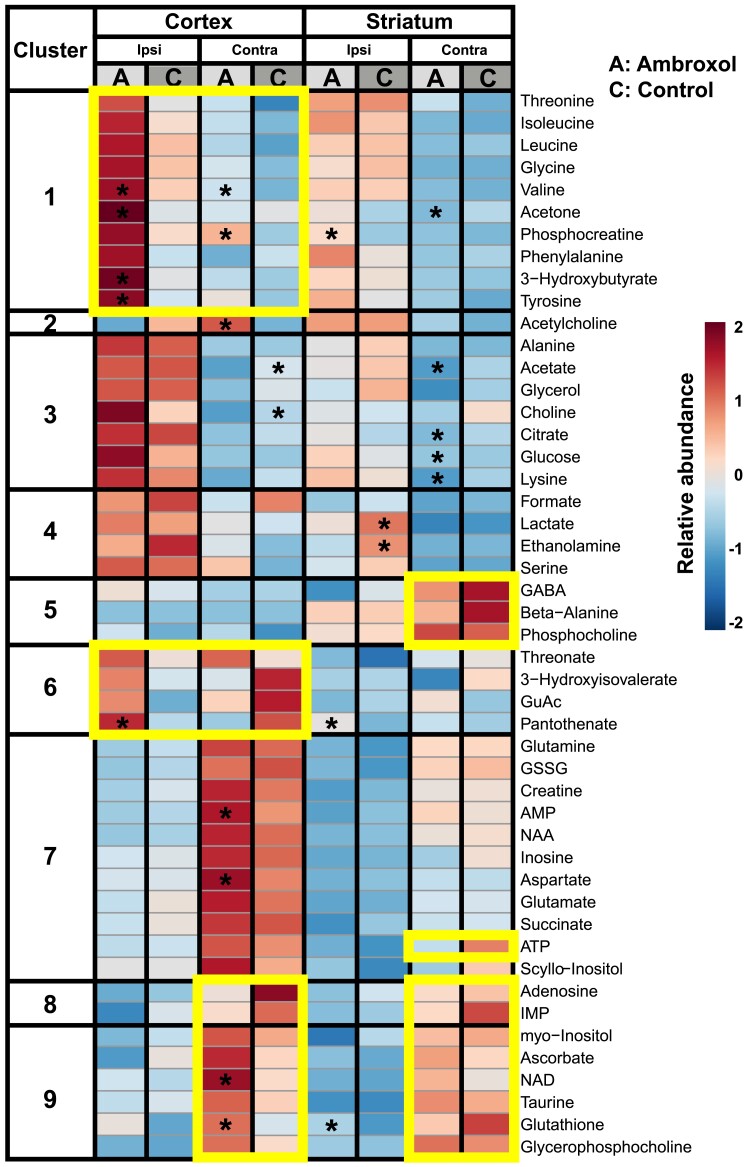
**Metabolomics data heatmap analysis (averaged by mean): ambroxol (A) over controls (C) in ipsilesional (Ipsi) and contralesional (contra) parts of rat striatum and cortex.** A total of nine similar metabolic clusters were identified via Ward’s method. The major metabolic differences (bright boxes) are discussed in detail in the Results and Supplementary sections. *t*-test comparisons were made between the control and ambroxol group, * *P*-value < 0.05. There were no significances found after FDR correction. GABA, gamma-aminobutyric acid; GuAc, guanidinoacetate; GSSG, glutathione disulfide; AMP, adenosine monophosphate; NAA, *N*-acetylaspartate; ATP, adenosine triphosphate; IMP, inosine monophosphate; NAD, nicotinamide adenine dinucleotide.

The contralateral cortex of the ambroxol-treated animals showed significantly higher concentrations of adenosine monophosphate (AMP), NAD, valine, aspartate, acetylcholine, and glutathione (*P* < 0.05), as well as lower levels of acetate and choline than those of control animals (Table 1 and Fig. 7). Further metabolites, such as phosphocreatine, glucose, and citrate, exhibited substantial fold changes (Table 1, cortex contralesional).

The cortical stroke region of ambroxol animals had higher concentrations of pantothenate (vitamin B5), valine, tyrosine, acetone, and 3-hydroxybutyrate compared to the control animals (*P* < 0.05). Moreover, in this region, ambroxol animals also show in average higher metabolic concentrations of aliphatic (threonine, glycine) and aromatic (phenylalanine, tyrosine) amino acids, branched-chain amino acids (BCAAs: isoleucine, leucine, valine), ketone bodies (acetone, 3-hydroxybutyrate), glucose, choline, and phosphocreatine in comparison to control animals. Additionally, the above-mentioned metabolites were considerably increased in comparison to the contralesional cortex as shown in Fig. 7.

The contralesional striatum in both animal groups showed decreased aliphatic, branched-chain, and aromatic amino acids, as well as ketone bodies. However, glucose, citrate, acetate, lysine, and acetone were significantly reduced in ambroxol rats compared to controls (*P* < 0.05, Table 1, striatum contralesional). Notably, GABA, beta-alanine and phosphocholine are increased in the region, with slightly higher average fold concentrations in controls, than in ambroxol animals (Fig. 7). Herein, adenosine triphosphate (ATP), adenosine, and IMP were also more abundant in control animals than in ambroxol animals.

Glucose and various amino acids were increased in the striatal stroke region of both animal groups, but most predominantly in ambroxol animals (Table 1, striatum ipsilesional and Fig. 7). Relative to the contralesional striatum, we identified increased metabolic concentrations of pantothenate (vitamin B5), phosphocreatine, and glutathione in ambroxol animals (*P* < 0.05). Importantly, the striatal stroke of ambroxol animals presents lower relative concentrations of lactate and ethanolamine in comparison to control (*P* < 0.05).

**Figure 8:**
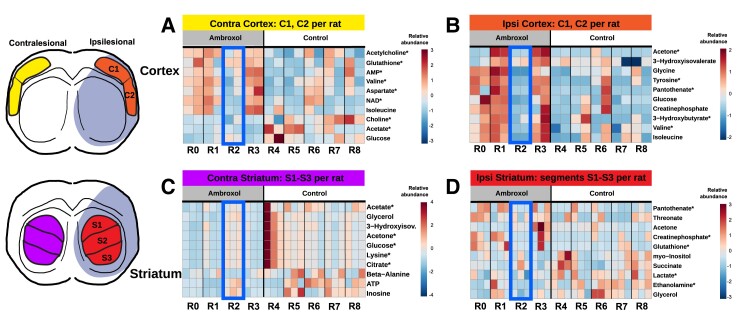
**Metabolomics data heatmap analysis plots.** Ambroxol over controls in: **(A)** contralesional (Contra) parts of rat cortex, **(B)** ipsilesional (Ipsi) parts of rat cortex, **(C)** contralesional (Contra) parts of rat striatum, **(D)** ipsilesional (Ipsi) parts of rat striatum. Statistical significance: * refers to *P* < 0.05. NAD, nicotinamide adenine dinucleotide; ATP, adenosine triphosphate; 3-Hydroxyisov, 3-hydroxyisovalerate.

The heatmap plots in Fig. 8 summarize the main effects per brain segment for every individual rat involved in the metabolomic analysis. We can observe that glutathione, acetylcholine, AMP, NAD as well as various amino acids (valine, aspartate) are more abundant in the contralesional cortex of ambroxol-treated animals than in controls (Fig. 8A). Interestingly, the ambroxol-treated animal R2 presented the smallest stroke of all animals in this analysis (Supplementary Table 6) and showed consistently different metabolite profiles to all its peers (blue box). The cortical stroke region of ambroxol in comparison to control animals showed strong increments in glucose, phosphocreatine, and various amino acids (glycine, valine, and isoleucine; Fig. 8B). In the contralesional striatum region, we found higher amounts of beta-alanine, inosine and ATP in controls compared to ambroxol animals (Fig. 8C). Finally, the striatal stroke displays overall a very similar metabolic profile between both groups, however, of relevance, intra-animal variance can be seen as a heterogeneous sampling of glutathione, creatine phosphate, and lactate, likely due to the difference in stroke volume (Fig. 8D).

## Discussion

In this work, we evaluated multiple aspects of IS in rats following ambroxol administration. In comparison to controls, ambroxol animals presented significantly reduced stroke volumes and milder cytotoxic oedema, specifically in the striatum during the sub-acute phase (24–72 h post-stroke onset). Volume reductions were accompanied by improved behavioural outcome as well as changes in structural and functional connectivity. *Ex vivo* NMR analysis additionally disclosed a metabolomic phenotype that supports increased free radical scavenging, retention of specific metabolites associated with cellular energy reserves, and abundant amino acid concentrations in ambroxol animals. Finally, in the chronic stroke phase (starting 1-week post-stroke onset), ambroxol treated animals presented significantly less white matter degeneration of the external capsule and less necrosis of the stroke area in comparison to control animals.

### The sub-acute stroke phase under ambroxol administration (24–72 h)

Motor and sensory deficits are the most common symptoms in stroke patients and are directly influenced by stroke volume and brain oedema.^58^ Our animal data is in line with this paradigm. Ambroxol animals presented smaller stroke volumes, specifically in the striatum with matching milder coordination and sensory deficits. Interestingly, rm-ANOVA showed also a significant stroke volume reduction in the cortex, which is a visually observable effect in the posterior regions of the brain above the hippocampus (Fig. 1A; Supplementary Table 4 index no. 3). Post-hoc testing however, did not resolve any significant comparisons. This observation suggests that ambroxol exerts stronger effects in the striatum than in the cortex. A possible explanation is that the striatum presents physiologically lower perfusion and anatomically less collateral vascularization than the cortex. Therefore, the stroke volume reduction effect of ambroxol in the cortex may be partly masked by a co-occurring physiological protection produced by available collateral blood flow. In contrast, the striatal regions do not enjoy this vascular rescue. Here, it is important to point out that the contralateral compensation in the cortex may not be so successful in humans due to chronic vascular disease. Therefore, the effect of ambroxol could be even more beneficial or at least more apparent in humans. These findings suggest that ambroxol can be used as an adjuvant after reperfusion to reduce stroke volume. The effects may improve functional outcome already in the acute phase in humans.

Cytotoxic oedema in the hyperacute and acute phases is followed by neuronal body death, which is the proposed precursor of axonal degeneration (Wallerian degeneration). Both groups presented similar cytotoxic and vasogenic oedema in the acute stroke phase in the striatum and cortex, although ambroxol animals were slightly less affected than controls. Neuronal death in the striatum and cortex is followed by axonal degeneration in the ipsilateral and contralateral corpus callosum.^59,60^ The corpus callosum consists of dense white matter fibre bundles responsible for relaying information between both brain hemispheres and is known to present impairments during stroke. We had hypothesized that ambroxol would aid in reducing neuronal necrosis, thus helping maintain white matter integrity in the corpus callosum fibres projecting from the striatum. In fact, both animal groups presented reductions of FA in the external capsule, however, the control animals continued to deteriorate into the chronic phase, while ambroxol animals begin to recover throughout the week of therapy. Interestingly, the 24 h timepoint presented the most prominent sensory and coordination behavioural deficits, but at this timepoint only the ipsilateral genu and internal capsule showed white matter alterations (Fig. 4D and E). Here, we found that the white matter degeneration of the internal capsule was actually significantly worse in ambroxol animals than controls at 24 h. Although the FA of the internal capsule of control animals continued to significantly and consistently decline over 1 week, the white matter deterioration of ambroxol animals did not progress. However, sensorial and coordination tasks showed significant differences first at 24 h and since internal capsule carries sensory and motor fibres;^61,62^ we expected a worsened FA at this timepoint for the control group. An explanation may be that the behavioural phenotype of controls at 24 h is not primarily related to the damage of the internal capsule, but to the prominent cytotoxic oedema of the neuronal bodies in striatum and cortex as well as the actual stroke volume. rs-fMRI evaluated the neuronal activation in these regions, which confirmed significant reductions of function in the motorsensory pathways at this exact time point. Moreover, there was white matter degeneration in the contralateral internal capsule and genu as well, which has been shown to play a role in sensory/motor phenotypes.^59^ Taking together the structural connectivity, cytotoxic oedema, stroke volume, and behavioural findings, we stipulate different co-occurring scenarios: ambroxol administration reduced striatal and cortical neuronal death at 24 h, which led to an improved behavioural outcome and functional connectivity, and ambroxol administration during 1 week aided at suppressing white fibre degeneration in multiple regions of the corpus callosum. We then aimed to gain further understanding using rs-fMRI and metabolomics.

rs-fMRI is used in preclinical and clinical studies for the detection of changes in functional brain connectivity in the whole brain. Our rs-fMRI data supports the hypothesis that ambroxol reduces overall signal transduction in the brain leading to reductions in global rs-FC by blocking Na^+^ channels and affecting Ca^2+^ homeostasis.^18,19,63^ These reduction in global connectivity for ambroxol animals are in line with changes in energy consumption observed in metabolic analysis. Stroke itself reduces global rs-FC, as previously shown in the acute and sub-acute phases.^64,65^ However, ambroxol-treated animals presented a notable homogeneous global reduction in rs-FC in comparison to the focal connectivity changes of the control group (Supplementary Fig. 4). In addition to global brain connectivity differences, we found relevant focal connectivity rs-FC differences in both animal groups at 24 h.

When taking into account rs-FC differences not corrected by FDR, the control animals presented a slight increment in connectivity between the ipsilesional striatum and the ipsilesional M1 and S1 regions, as well as between the contralesional striatum and the ispilesional S1 regions compared to ambroxol (Fig. 5). These differences were also observed when comparing baseline to 24 h measurements between both groups (Fig. 6). As previously addressed, we hypothesize that this occurs at least partly, due to the more severe cytotoxic oedema at 24 h for control animals in the striatum. Moreover, the increased contralesional striatum activity in control animals may be compensatory due to the reduced sensorimotor output of the ipsilesional striatum. However, this potential compensation, is not successful since the sensory and coordination behavioural tests of controls show nevertheless more severe deficits at 24 h than ambroxol animals. Additionally, since the contralesional external capsule (Supplementary Fig. 3B) is less affected than the ipsilesional external capsule, contralateral connectivity should be structurally possible.

From another point of view, in general, the slightly increased functional connectivity evaluated in controls may reflect more prominent neuroinflammation in control animals, which the BOLD signal reads out as increased connectivity correlations. This remains unclear and requires further specifically dedicated research to be completely understood and deconvoluted. Moreover, further fMRI evaluations with a larger animal number and a different choice of anaesthetic, such as medetomidine + isoflurane^66^ may yield more conclusive results after FDR correction. Regardless, our group comparisons demonstrate that ambroxol administration is associated with significant changes in regional blood flow of the stroke cortex. Importantly, it was not delimited to the motor and sensory ipsilateral regions, but also showed effects in contralateral brain regions, consistent with structural white matter changes.

The general effects of stroke on brain tissue in our metabolomics data are in accordance with previous investigations. Stroke drug treatments were characterized before by reduction of oxidative stress, lowering inflammation, and recovering energy metabolism.^67,68^ Positive metabolite alterations after drug treatment in stroke models have been reported for glucose, lactate, ATP, and glutathione.^69,70^ Other metabolites such as NAA have a complex interpretation. NAA has been suggested as a biomarker of neuronal activity and neuronal density^71^ as well as an indicator of neuronal injury.^69^ NAA has been found reduced in the stroke-affected rat brain, consistent with our results.^68–70^ However, evaluating NAA improvement to determine stroke severity and response to therapy may be constrained.^72,73^ Therefore, it may be helpful to assess other upregulations during ischaemia, e.g. levels of energy metabolites and antioxidants.

In our study, enhanced energy and redox metabolism was observed in ambroxol-treated rats. Hereby, the cortical region in ipsilesional hemisphere samples displayed metabolite changes that play a supporting role in healthy brain metabolism (glutathione is an antioxidative metabolite, pantothenate is vitamin B5, acetone and 3-hydroxybutyrate are ketone bodies, which are an important energy source when glucose drops e.g. under starvation, Table 1, cortex ipsilesional). Higher levels of pantothenate were shown to stabilize the antioxidative response from glutathione.^74^ Pantothenate is further a precursor for both acetyl-coenzyme A (acetyl-CoA, an essential cofactor in cells) and acetylcholine.^75^ Choline, acetylcholine and ethanolamine sustain cell membrane homeostasis and are linked to energy metabolism during IS^76,77^ Therefore, in an excess of pantothenate, brain tissue cells may be able to utilize these metabolites for repair. Moreover, higher levels of tyrosine are associated with higher generation rates of ROS scavengers.^78^ In addition, phosphocreatine (2.3-fold increase, Table 1, cortex ipsilesional), a metabolite which is important during ischaemic events and supports neuronal activity,^79^ was markedly increased in ambroxol animals (Fig. 8B and D). As a result, significant amounts of ketone bodies in ambroxol animals (up to 3-fold greater consumption in the cortex, Table 1, cortex ipsilesional) might be utilized as an energy source for stroke-affected brain regions in the absence of glycolytic energy metabolites, thereby benefiting stroke therapy.^80^ Valine and isoleucine are known to influence glutamate regulation,^81^ were also increased in ambroxol animals (Fig. 7). The increased concentrations of phenylalanine and tyrosine together with increased BCAAs are vital for sustaining protein stability in the brain tissue. Additionally, the exclusive increment of aminoacids, specifically tyrosine in the cortex of ambroxol animals may be associated to ambroxol’s effects on tyrosine metabolism.^17^ Summarizing, animals administered with ambroxol presented increased BCAAs levels in the cortex, ketone bodies, phosphocreatine and tyrosine (Fig. 7 and Table 1). In a combination with higher deposits of glucose and pantothenic acid, three out of four ambroxol-treated rats likely underwent a recovery process taking place in cortical tissue of the ipsilesional hemisphere. Moreover, a clear difference in metabolite concentrations can be observed in the ambroxol animal with the smallest stroke volume in Fig. 8.

The contralesional cortex showed further increments in antioxidative metabolites as well as a significant increase in the energy deposit molecule phosphocreatine in ambroxol animals. Contralesionally, ambroxol animals showed remarkably lower pantothenate levels (2.8-fold, Table 1, cortex contralesional), completely the inverse of the control animals, suggesting that pantothenate could be redirected to the ipsilesional hemisphere only in ambroxol treated animals. This mechanism has been described for acetylcholine, secreted from cholinergic neurones in the striatum to protect tissue during stroke.^82^ At the same time, our data shows that ambroxol animals presented significantly higher acetylcholine in the contralesional cortex than controls. ROS ischaemic damage metabolic markers in the ambroxol group rats also became highly concentrated in the contralesional cortical segments along with higher AMP, aspartate, and NAD compared to controls. Herein, the described ambroxol-defined change in tissue metabolome of the contralesional rat cortex suggests a possible interconnection with the stroke hemisphere by undergoing degradation of phospholipids in cellular membranes, energy availability, and increasing ROS formation. In fact, each rat segment of all the ambroxol group rats developed a higher glutathione supply (Fig. 8A). Therefore, our results suggest an effect of ambroxol in ROS regulation and increased availability of acetylcholine consistent with an improved stroke outcome.

The increments of glutathione, ATP, pantothenate, as well as the reductions of lactate and citrate in the ipsilesional striatum of ambroxol animals are consistent with a neuroprotective effect in the context of the neuroimaging results and behavioural data of the current study. Moreover, lower levels of glycerol and significantly lower amounts of ethanolamine (2.1-fold decrease, Table 1, striatum ipsilesional) could be also attributed to the positive effects of sustaining phospholipid content in cellular membranes. Increased phosphocreatine in striatal tissue of the treated rats could provide an additional source of phosphate for the damaged neuronal tissue. The contralesional striatum of both animal groups presented a lower relative abundance of BCAAs, with significantly lower citrate, glucose, lysine, acetate, and GABA on the ambroxol animals as control animals. The GABA concentrations are particularly interesting, since they are increased only in the contralesional striatum of both animal groups in comparison to the contralateral cortex, the ipsilesional striatum and cortex. This finding could be interpreted as an effect of the stroke-free region compensating for the lack of the neurotransmitter in the interconnecting regions (Fig. 7), which is consistent with the significantly increased functional connectivity of control animals between contralateral striatum and the ipsilateral sensory cortex found at 24 h (Fig. 5B, 24 h). The GABA concentrations are notably less for ambroxol than control animals, herein, the metabolomics results align with the results of the fMRI-based evaluation of global functional connectivity at 24 h.

Taking altogether, the metabolomics data supports that ambroxol administration provoked substantially positive metabolic effects towards recovery of striatal and cortical tissue.

### Long-term effects of sub-acute ambroxol therapy in chronic stroke (1 week to 1 month)

Regions with severe cytotoxic oedema at 24 h transform to infarcted areas when cells lose their membrane integrity resulting in cyst formations or necrosis.^83^ Liquefaction of the necrotic tissue presents higher free water diffusivity in those fluid-rich cystic regions,^29,31^ evidenced by increased ADC and T2W signal intensity at 1 month (Fig. 2). Besides, less free-diffusion in the stroke region, ambroxol animals showed improved FA values after 1 week and 1 month in the ipsilesional external capsule. The recovery of axonal integrity at 72 h and 1 week after stroke onset on ambroxol animals is consistent with previous reports of FA return to baseline after 3 weeks if no permanent damage occurs to the axons.^36,84^

It was suggested that the contralesional M1 cortex may play an important role for motor recovery within the first 4 weeks after stroke onset.^85,86^ Consistently, a significant reduction of rs-FC between ipsi- and contralesional striatum was found in both groups at 1 month, suggestive of a persistent connectivity deficit (Fig. 6). However, a direct look at the motor pathway (Fig. 5) at 1 month shows higher increments in rs-FC at 1 month for controls in comparison to ambroxol animals. This finding denotes that control animals still require contralateral rs-FC increments at 1 month, while ambroxol animals do not. Both groups present similar behavioural recovery after 1 month, however the networks leading to this outcome appear to be partly compensatory for controls, but not for ambroxol. However, this effect is observed in data before FDR correction; therefore, it should be interpreted cautiously.

### Study limitations and recommendations

Our work presents some limitations, which we would like to address in order to improve further preclinical therapeutic trials. We did not experiment on ‘sham’ animals, or animals without a stroke, for the behavioural analysis or for metabolomic analysis. In behavioural experiments, this option would have allowed us to better interpret the behaviour of stroke animals over time, in the framework of the normal habituation observed in ‘sham’ animals. Moreover, metabolomic analysis could have benefited from healthy brain tissue as control, instead of using contralesional brain tissue. As our evidence demonstrates, the contralesional brain is involved in compensating ipsilesional functional deficits in a macro and metabolic level. Therefore, we recommend the inclusion of sham animals in similar studies. In addition, although blinding was performed for all data analysis and data acquisition, the experimenters were not blinded during the daily intraperitoneal administrations of NaCl, PEG or ambroxol. Therefore, bias in animal handling during drug administration cannot be ruled out. However, our longitudinal evaluation of the NaCl and PEG control groups showed that both control groups are identical, which reflect the consistency and robustness of the performed experimental procedures (Supplementary Table 3).

Regarding the statistical findings of the metabolomics data and functional connectivity; these methodologies produce an exceptionally large number of features to be evaluated from a single biological replicate. This leads to a particularly high number of comparisons, which in turn makes FDR thresholding considerably conservative. We show *P*-values with and without FDR correction in order to disclose all findings, however future investigations should increase the number of samples allocated to these demanding methodologies.

Our work combines an advanced framework of neuroimaging techniques with dedicated behaviour evaluations and *ex vivo* metabolomics analyses. This approach provides a complex insight into the neuropathophysiology of stroke and the metabolic and functional mechanisms involved in neuroprotection and recovery from the sub-acute to the chronic phase. Our data shows that ambroxol-administered rats presented significantly smaller and less severe oedematous stroke lesions, improved behavioural outcome, as well as congruently altered functional and structural connectivity. Specifically, this early reduction of stroke volume and oedema induced by ambroxol, when started shortly after reperfusion, may clinically reduce mortality and morbidity. Furthermore, our metabolomics investigation hints at beneficial metabolic changes in the brain of animals treated with ambroxol. These changes are mainly characterized by elevations of antioxidants and ketogenic energy metabolism and a metabolic phenotype consistent with reduced neuronal damage. In the chronic stroke phase, our neuroimaging findings demonstrate less necrotic stroke regions, as well as better preserved white matter integrity. To our knowledge, this is the first report of ambroxol as a possible neuroprotective agent for IS.

## Supplementary Material

fcad099_Supplementary_Data

## Data Availability

The data presented in this manuscript can be made available upon reasonable request to the authors.

## Abbreviations

acetyl-CoA =: acetil-coenzyme A
ADC =: apparent diffusion coefficient
AMP =: adenosine monophosphate
BCAA =: branched-chain amino acid
BOLD =: blood-oxygen-level-dependent
CPu =: corpus putamen
DWI =: diffusion weighted image
EC =: external capsule
FA =: fractional anisotropy
FDR =: false discovery rate
fMRI =: functional MRI
GSSG =: glutathione disulfide
GuAc =: guanidinoacetate
IC =: internal capsule
IMP =: inosine monophosphate
IS =: ischaemic stroke
M1 =: primary motor region
S1 =: somatosensory region
NAA =: N-acetylaspartate
NaCl =: sodium chloride
NAD =: nicotinamide adenine dinucleotide
NMR =: nuclear magnetic resonance
OC =: occlusion
PEG =: polyethylene glycol 400
ROS =: reactive oxygen species
rm-ANOVA =: repeated measures ANOVA
rs-FC =: resting-state functional connectivity
rs-fMRI =: resting-state functional MRI
SD =: standard deviation
SI =: signal intensity
T2W =: T2-weighted
TCA =: tricarboxylic acid or Kreb's cycle
Th =: thalamus
VOI =: volumes of interest
