# Supplementary material for: Repurposing the mucolytic agent ambroxol for treatment of sub-acute and chronic ischaemic stroke
Source: Brain Commun. 2023 Mar 29;5(2):fcad099. doi: 10.1093/braincomms/fcad099 (PMC10090797; doi:10.1093/braincomms/fcad099)
Supplement: fcad099_Supplementary_Data [file fcad099_supplementary_data.pdf]

Repurposing the mucolytic agent Ambroxol for treatment of sub-acute and chronic ischemic stroke

Kristin Patzwaldt[1], Georgy Berezhnoy[1], Tudor Ionescu[1], Linda Schramm[1], Yi Wang[2], Miriam Owczorz[1], Eduardo Calderón[3], Sven Poli[2], Lina M Serna Higuita[4], Irene Gonzalez-Menendez[5,6], Leticia Quintanilla-Martinez[5,6], Kristina Herfert[1], Bernd Pichler[1,6], Christoph Trautwein[1]*, Salvador Castaneda-Vega[1,3]*

Supplementary Materials

Supplementary Methods

In supplementary Table 1 and 2 all animal numbers for imaging and behavioral experiments are listed for the different time points.

MCAO stroke surgery

Ischemic stroke was induced on the right hemisphere using the temporal Middle Cerebral Artery Occlusion (MCAO) surgery for 60 min as described previously by Longa et al. [1] and as we have previously established in our laboratory with modifications [2]. In short, surgery was performed under isoflurane applied with a face mask, which was vaporized in room air with a flow rate of 0.8 l/min. A surgical microscope was used for performing the surgery. After disinfection and analgesics application, surgery was started with a mid-line incision on the neck. The common carotid artery (CCA), the internal carotid artery (ICA) and external carotid artery (ECA) were dissected and surgical clamps were placed on the CCA and the ICA to avoid retrograde bleeding. To insert the silicon-coated nylon filament (4-0 nylon; Ø = 0.41 mm, 3 - 4 cm length; Doccol Corporation, USA) an incision on the ECA was made. After fixating the filament in the ECA stump with a suture, it was pushed in the CCA bifurcation and further into the ICA. Next, the first clamp around the ICA was loosened allowing the filament to be pushed further into the skull, up to the MCA. As soon as the filament was placed correctly, the second clamp around the CCA was loosened and the wound was closed temporarily. Occlusion was maintained for 60 minutes during which time MRI measurements were performed to confirm lesions in diffusion weighted images (DWI). Immediately after the occlusion time was over, the filament was removed and reperfusion into the carotid artery was restored. The surgical wound was closed permanently. Rats received post-surgery pain treatment of carprofen (5mg/kg, Rimadyl®, Zoetis Schweiz GmbH, Switzerland) up to 3 days.

Animal handling, numbers and distribution

For our experiments a total number of 73 rats were used weighting between 324 g and 428 g (median: 356 g) on the day of surgery. Animals were kept in groups of 3 to 4 animals per cage with wooden enrichment next to the wood chip embedding and nesting material. Animals were fed with 15 g of standard food pallets per day and per animal before surgery and had *ad libitum* access to food after surgery. Furthermore, *ad libitum* access to water was ensured all the time. Room temperature was kept at 22°C (± 1°C) and humidity was constantly held around 57% (± 5%) with a day-night cycle of 12 hours.

From all planned animals only 53 were further evaluated for analysis. In total 20 animals were not analyzed. Seven animals did not present a successful MCAO stroke induction and were not further included in the study. One animal presented imaging artifacts that led to insufficient quality ADC and T2W images for analyses. Twenty animals in total were excluded from analysis. From these 12 animals were excluded since no longitudinal imaging data was available; they died during or shortly after surgery. Out of those 12 animals, five animals died peri-surgical to the MCAO, either during or shortly after surgery, before therapy administration. One animal that received one dosage of NaCl treatment died within one hour after surgery. Three animals that received one dosage of PEG treatment died within one hour after surgery. Three animals that received one dosage of Ambroxol treatment also died; 2 within one hour after stroke and one died during the 24 h MRI scan.

Following ethical guidelines, animal behavior, fur, weight, and adequacy of self-feeding among other criteria were monitored and evaluated daily for a week after stroke and then at least at three times per week until the endpoint.

Treatment

The injection volume was calculated so that the administered amount of liquid for each injection did not exceed the prescribed capacity of 10 ml/kg by the GV-SOLAS. The injected amount of Ambroxol for the therapy group accounted for 45 mg/kg per treatment for a total Ambroxol amount of 90 mg/kg per day and per animal. The NaCl control group received NaCl only with the same amount of liquid per injection as the Ambroxol therapy group. The PEG control group received a solution consisting of NaCl and PEG each partial of 50% with the same amount of liquid per injection as the Ambroxol therapy group.

MRI data acquisition

Acquisitions were performed in a 7 T BioSpec MRI with a B-GA12S HP gradient insert using a volume mouse whole body transmitter-receiver coil that covered the whole brain (Bruker BioSpin, Ettlingen, Germany). All animals were scanned at multiple time points from baseline, during occlusion as well as 24 h, 72 h, 1 week and 1-month post-surgery. Anesthesia was induced using isoflurane (2%-5%) vaporized in room air at 0.8 l/min. For fMRI measurements, rats were anesthetized with only 2% to keep

isoflurane influence as low as possible. During measurement, isoflurane concentrations were reduced. Animals were placed on an MR compatible water heated bed (Bruker BioSpin, Germany) where temperature was monitored and constantly kept at 37 ± 0.5 °C using a rectal temperature probe and a feedback heating system (Medres – medical research GMBH, Germany). An additional heating mat was fixed on top of the animals to prevent them from cooling down. The respiratory rate was monitored during MRI scans. Two different imaging protocols were used for the MRI scans: one protocol during occlusion and another protocol for all other time points. T2 weighted images (T2W) were acquired using a 2D-spoiled turbo RARE spin echo sequence (256 × 256 matrix, 35 x 35mm^2 field of view (FOV), repetition time (TR) = 8414.36 ms, echo time (TE) = 33 ms, slice thickness = 0.5 mm, 80 slices, averages = 3). During occlusion following parameters were different: TR = 4206.78 ms, TE = 11 ms; slice thickness = 0.8 mm and 40 slices. DWI were acquired at all time points using an echo planar imaging (EPI) diffusion tensor image (DTI) sequence on the coronal plane (128 x 80 matrix, 25 x 30 mm^2 FOV, TR = 2000 ms, TE = 22.13 ms, Flip angle = 90°, slice thickness = 0.8 mm, b-values = [400, 600, 800, 1000] s/mm^2, 30 diffusion directions and 30 slices). The first 15 animals were measured with 6 diffusion directions during occlusion in order to reduce scan time. Due to a lower signal to noise ratio, the protocol was changed to 30 directions during occlusion time for all other animals. Functional MRI (fMRI) images were acquired at baseline, 24 h and 1 month post-surgery using T2*-weighted gradient echo EPI sequences (92 x 59 matrix, FOV = 25 x 23 mm^2, TR = 2000 ms, TE = 18 ms, Flip angle = 90°, slice thickness = 0.8 mm, 20 slices) together with T2 anatomical reference scans (128 x 160 matrix, 40 x 32 mm^2 FOV, TR = 1800 ms, TE = 67.11 ms, 128 slices, averages = 1).

Image data pre-processing – ADC, FA and T2W images

ADC maps and FA maps were calculated from DWI using MATLAB (version R2019b, The MathWorks, Inc., Natick, USA) as well as ParaVision software (version 6.1.1, Bruker BioSpin) correspondingly. All T2W, ADC and FA images from all animals and all time points were co-registered and spatially normalized to the Schiffer rat brain atlas [3] using Pmod software (version 3.2 Pmod Technologies LTD., Zurich, Switzerland). The ipsi/contralesional ratio was also calculated per animal.

FA maps – T-test maps

T-test maps were calculated voxel wise between the two groups using Matlab with an alpha of 0.05. For further analysis, the corpus callosum VOIs were overlaid with each t-test map to quantify the percentage of significant t-test voxels in the corresponding region between the groups. Therefore, the volume of each VOI was divided by the volume of the significant voxels.

fMRI data preprocessing

In short, Statistical Parametric Mapping 12 (SPM 12, Wellcome Trust Centre for Neuroimaging, University College London, London, United Kingdom) was used to realign all fMRI scans and generate average images for each subject. Together with the anatomical reference scans, these average images were used to remove extra-cerebral tissues from all datasets employing binary masks. This step was

performed with Analysis of Functional NeuroImages (AFNI, National Institute of Mental Health, Bethesda, Maryland, USA). The skull-stripped fMRI scans were co-registered to their respective anatomical references. The anatomical scans were then employed to spatially normalize all scans to the Schiffer brain template. Afterwards, nuisance removal was performed using a multiple linear regression model including the six motion parameters generated during realignment, as well as principal components extracted from extra-cerebral tissues, as described by Chuang et al. [4]. For single subjects coregistration was further optimized manually. To smooth the fMRI data spatially a 1.5 x 1.5 x 1.5 mm^3 full-width-half-maximum Gaussian kernel was applied to all datasets [5].

fMRI data analysis and Brain regions

The brain regions defined by the Schiffer atlas in the Paxinos space [3,6] were extracted from fMRI data using the SPM toolbox Marseille Boîte À Région d'Intérêt (MarsBaR)[7] and are summarized in Supplementary Table 7. The time-courses were used to calculate pairwise Pearson's r correlation coefficients for each dataset, generating correlation matrices of 52 x 52 elements. Self-correlations were set to zero and computed Pearson's r coefficients were transformed into z values using Fischer's transformation and used to calculate group-level average matrices. To investigate potential effects between the different treatment groups, rs-FC metrics for the motor network (motor cortex, thalamus, sensorimotor cortex and striatum) and whole brain network (all 52 regions mentioned above) were computed on different spatial levels. The strength of a network was defined as the sum of strengths of all correlations between the networks' regions. Reductions of the z-transformed Pearson's r values over time were calculated in percent for individual correlations (e.g. ipsi- and contralesional striatum).

Behavioral experiments

The evaluations were performed before treatment administration and before MRI measurements to avoid effects of narcosis at 24 h, 72 h, 1 week and 1 month post stroke induction. All experiments were performed by the same evaluator in a dedicated sound-proofed behavioral room illuminated by central bulb. Rats were introduced separately into the room for every evaluation. A period of one week of habituation to the animal facility was allowed prior to start of baseline training. The total number of animals available for behavior and the number of animals for each test at each time point is summarized in Supplementary Table 2.

Beam Walk

The beam walk test measured the motor function of the animals by crossing a wooden beam. Before testing, animals had to be trained for 4 days before baseline measurement to cross the 60 cm long, 40 cm high and 1.7 cm wide beam. For each training and later measurement time point, animals had to cross the beam 5 times. All experimental trials were videotaped and started when all 4 paws of the animal were on the beam, and ended, when all paws were off the beam. In between the trials, the animals could rest in a cage which was placed at the end of the beam. A trial was stopped if an animal did not cross the beam within 1 minute. Together with the failed steps, counted data was evaluated

with a score: 0 = no failed steps, 1 = maximum of 2 failed steps, 2 = maximum of 5 failed steps, 3 = hangs off the beam but only one time or a maximum 10 failed steps, 4 = hangs off the beam several times or a maximum of 20 failed steps, 5 = cannot walk anymore but stays on the beam and 6 = falls off the beam. In addition, the successful crossing of the beam was counted. Trials of animals which did not finish within 1 minute because of lack of interest were removed from analysis. Animals were placed back into their cage after the measurement. At baseline, one animal was removed from the analysis because it did not complete any of the 5 iterations.

Sticky Label

The sticky label test measured the sensory and motor function of the animals. The premise of this test is that animals present paresthesia of an affected forepaw due to the stroke lesion. The sticky tape is therefore more difficult to be removed from the contralateral paw. A black colored sticky tape (approx. 1.2 cm x 1.2 cm, 3M Transpore™) was stuck to each forepaw of the animals. The animals were placed into a squared arena (47 cm x 47 cm x 26 cm) and videotaped while removing the tape. The test was aborted if the animal was not able to remove the tape after 2 minutes. Time and success rate of tape removal were quantified. Animals were placed back into their cage after the measurement. Animals not being able to remove the tape at baseline and any later time point (n = 3) were excluded from analysis.

Open Field

The open field test measured the explorative behavior and anxiety of the animals. Animals were placed into the squared open field arena (47 cm x 47 cm x 26 cm). The distance walked was recorded by light sensors around the arena using ActiMod (TSE Technical & Scientific Equipment GmbH, Bad Homburg vor der Hoehe, Germany). A habituation time of 1 minute was followed by a 10-minute measurement time. Animals were placed back into their cage after the measurement. Incomplete measurements due to technical software failure were excluded at different time points (see Supplementary Table 2).

Grip Test

The grip test measured the strength of the animals' paws. Animals were placed with all paws on a grid (Grip test BIO-GS3, Bioseb, Florida, USA) and were then pulled back gently from the grid with steady force till the animals released the grid. Each animal had to repeat the test 5 times for each time point. The maximal force for each timepoint and animal was determined. A recovery rate was calculated using the baseline value as a reference. Animals were placed back into their cage after the measurement.

***Ex vivo* NMR metabolomic analysis**

Chemicals

Solvents for the two-phase extraction were methanol (MeOH, LC-MS grade, CAS: 67-56-1) and chloroform ($CHCl_3$, CAS: 67-66-3) purchased from Sigma Aldrich (Sigma-Aldrich Chemie, Taufkirchen, Germany); and also, the ultrapure AccuGENE® Molecular Biology Water (CAS: 7732-

18-5) was purchased from Lonza, Verviers, Belgium. Phosphate buffer for the NMR metabolomics sample preparation was prepared with the use of 3-(trimethylsilyl)propionic-2,2,3,3-d_4 acid sodium salt (TSP, CAS: 24493-21-8), potassium phosphate dibasic (K_2HPO_4, CAS: 7758-11-4), sodium azide (NaN_3, CAS: 26628-22-8), hydrochloric acid (HCl, CAS: 7647-01-0), and deuterated water (D2O, deuterium oxide, CAS: 7789-20-0) - purchased from Sigma Aldrich (Sigma-Aldrich Chemie, Taufkirchen, Germany); and also potassium hydroxide (KOH, CAS: 1310-58-3) purchased from Roth (Carl Roth, Karlsruhe, Germany).

Sample preparation equipment

Covaris CryoPrep™ pulverization system was used for tissue pulverization. The system and tissue bags for pulverization were purchased from Covaris Inc., Woburn, USA. Sartorius Ionization vent (220V) was used for efficient tissue powder aliquot preparation. Adaptive focused acoustics (AFA™) ultrasound technology Covaris E220 Evolution (Covaris, Inc., Woburn, USA) was used for sample homogenization and metabolite extraction. 2 mL AFA™ glass tubes were purchased from Covaris Inc., Woburn, USA. Ultrasonication program setup: two treatment cycles, 1st: 30 sec, Peak Power 125.0, Duty Factor 32.0, Cycles/Burst 400, Avg. Power 40.0. 2nd: 30 sec, Peak Power 100.0, Duty Factor 30.0, Cycles/Burst 800, Avg. Power 30.0. Temperature range 5.0 to 15.0°C. Each cycle repeated 5 times per sample, total run time per sample is 5 min. Christ Alpha 2-4LDplus lyophilizer was used for powder dry freezing (Martin Christ Gefriertrocknungsanlagen, Osterode am Harz, Germany). Savant™ SpeedVac™ SPD300DDA vacuum concentrator (Thermo Fischer Scientific Inc., Asheville, USA) was used for extraction solvent evaporation to dryness. SpeedVac set-up used: *Preset 2* (low boiling MeOH/ACN). Temperature: 45°C, heat time: 2 h, run time (total including heat time): 2 h, vacuum level: 100 mTorr, Vacuum ramp: 5. Centrifuge Thermo Fischer Scientific Heraeus Megafuge 8R was used as part of liquid-liquid phase and liquid-solid phase separation (Thermo Electron LED, Osterode am Harz, Germany). Prior to the NMR sample preparation, water-phase extracts were homogenized using an Elma® S 60H Elmasonic sonicator (Elma Schmidbauer, Singen, Germany).

Metabolite extraction procedures

Tissue samples were initially pulverized at Impact level 1, then were transferred to 2 mL AFA™ Covaris glass tubes. In tubes, samples were mixed well with 900 µL of MeOH, 100 µL of $CHCl_3$, and 100 µL of ultrapure water with the use of vortexing and were later loaded in the Covaris ultrasonicator. After extraction, solutions were loaded into a centrifuge (12000 x g force, 10 min) for complete phase separation. Centrifuged samples were later manually decanted with a mechanical pipette. The chloroform layer was transferred to an HPLC vial, while the polar phase was placed into an Eppendorf cup. Polar phase solutions were evaporated to dryness overnight with the vacuum concentrator (SpeedVac: Preset 2). Dried pellets of polar phase solutions were resuspended in 45 µL of deuterated phosphate buffer (200 mM K_2HPO_4, 200 µM NaN_3, pH 7.4) with 1 mM internal standard TSP.

Eppendorf cups containing solutions were sonicated for 1 min before being centrifuged for 5 min at 14,000 g to remove any solid residue. Volumes (40 µL) of the supernatant were put into 1.7 mm NMR tubes using gel loading pipette tips and then placed in a sample rack. Samples were kept cool (6°C) in the SampleJet until the spectra measurement.

Spectral data acquisition

NMR spectra were recorded by a 14.10 Tesla (600 MHz for proton channel) NMR spectrometer Avance™ III HD with the 1.7 mm probe PA TXI 600S3 H/P-C-D-01.7 Z (Bruker BioSpin, Fällanden, Switzerland). The spectrometer has an automation module Bruker SampleJet (Bruker BioSpin, Fällanden, Switzerland), an automation unit that allows sample queuing and sample cooling (6 - 10°C) maintained. Carr-Purcell-Meiboom-Gill (CPMG) experiment was used for the metabolomics analysis, with background signals from residual macromolecules suppressed (time domain = 65536 points, sweep width = 20 ppm, number of scans = 512-2048). The recorded free induction decays (FIDs) were Fourier-transformed (FT); phase and baseline corrections were performed.

Data evaluation

For spectra acquisition and processing, Bruker TopSpin 3.6.1 software (Bruker BioSpin, Rheinstetten, Germany) was used (offset correction, baseline and phase correction). ChenomX NMR Suite 8.5 Professional (Chenomx Inc., Edmonton, Canada) was used for metabolite annotation and concentration calculation. Internal Chenomx version 11 and the Human Metabolome Data Base (HMDB, http://www.hmdb.ca) Release 2 library were utilized for the dataset at the resonance frequency of 600 MHz. A concentration table was then exported from ChenomX NMR Suite as a comma-separated file and further used for statistical analysis. MetaboAnalyst 5.0 R-based online analysis tool (www.metaboanalyst.ca) was used for the statistical analysis of metabolomics data. Metabolites were excluded if more than 66 percent of the results for a metabolite were missing. Aside from that, missing values were replaced with a small value (1/5 of the minimum positive value of each metabolite in the original data). The data was normalized by a reference sample using probabilistic quotient normalization (PQN) to account for dilution effects [8] and scaled using Pareto scaling (mean-centered and divided by the square root of the standard deviation of each variable). Hierarchical clustering (Euclidian distance and Ward's linkage clustering) was used to produce heatmap plots of metabolite concentration changes across different groups. The three striatum portions were pooled together per animal group. The two cortical regions were also evaluated together.

Histology

After decapitating the animals, brains were removed, frozen, and cut in 5, 10 and 20 µm thick slices. For haematoxylin and eosin (H&E) staining brains were fixed for 5 minutes each in formalin and distilled water and afterwards stained with an H&E protocol in collaboration with the dermatology laboratories of the university hospital of Tuebingen (control n = 7; Ambroxol n = 4). Immunohistochemistry staining was performed on one animal per group by the pathology institute of

the university hospital of Tuebingen. The frozen sections were fixed in 4% formalin for 30 min (for cleaved Caspase 3) or overnight (for H&E and GFAP). Immunohistochemistry was performed on an automated immunostainer (Ventana Medical Systems, Inc., Arizona, USA) according to the company's protocols for open procedures with adapted modifications using the following antibodies: GFAP (glial fibrillary acidic protein, 6F2, Dako Deutschland GmbH, Germany); cleaved Caspase 3 (ASP 175; Cell Signaling Technology, Frankfurt am Main, Germany). Appropriate positive and negative controls were used to confirm the adequacy of the staining. All samples were scanned with the Nanozoomer (2.0-HT Hamamatsu Photonics K.K., Hamamatsu, Japan; 40x zoom) and processed with the programs Case Viewer (version 2.2, 3DHISTECH, Budapest, Hungary) and Adobe Photoshop CS6 (version 13.0.1, Adobe Systems Software Ireland Limited, Dublin, Ireland).

Supplementary Results and Discussion

Normalized T2W signal intensity, and apparent diffusion coefficient (ADC)

Supplementary Figure 1A presents the ratio for T2W over time for both groups. The ratio was calculated by dividing the ipsilesional hemisphere by the contralesional hemisphere. T2W values did not show any changes at baseline and occlusion. The reductions found from baseline to occlusion were due to reduction of the absolute T2W values (Fig. 2B, 2D and 2F) since we performed a shorter version of the sequence for anatomical positioning during occlusion. A main group effect was found by rm-ANOVA for the ratio of the complete stroke volume ($p = 0.011$, Supplementary Table 4 index no. 7) and striatal stroke volume ($p = 0.023$, Supplementary Table 4 index no. 8) but not for the cortical stroke volume ($p = 0.346$, Supplementary Table 4 index no. 9). At 1 month, significant differences between the groups were found for the ratio of the complete ($p < 0.001$) and striatal SV ($p < 0.001$) consistent to absolute values.

The ratios for the ADC values (Supplementary Figure 1B) were around 1 at baseline and decreased during occlusion, in the ipsilesional hemisphere. The ratio normalized over time reaching a ratio of around 1 between 72 h and 1 week for both groups [9]. A significant difference between control and Ambroxol can be seen at 1 month post stroke, consistent with absolute quantification. The control group presented a higher ratio of ADC in the whole stroke region ($p < 0.001$) and in the subset analysis of striatum ($p < 0.001$). A main group effect was found by rm-ANOVA for the ratio of the complete stroke volume ($p = 0.040$, Supplementary Table 4 index no. 13) and striatal stroke volume ($p = 0.028$, Supplementary Table 4 index no. 14) but not for the cortical stroke volume ($p = 0.684$, Supplementary Table 4 index no. 15). These results underlie the findings discussed already in the current study, demonstrating no differences could be observed in the contralesional hemisphere.

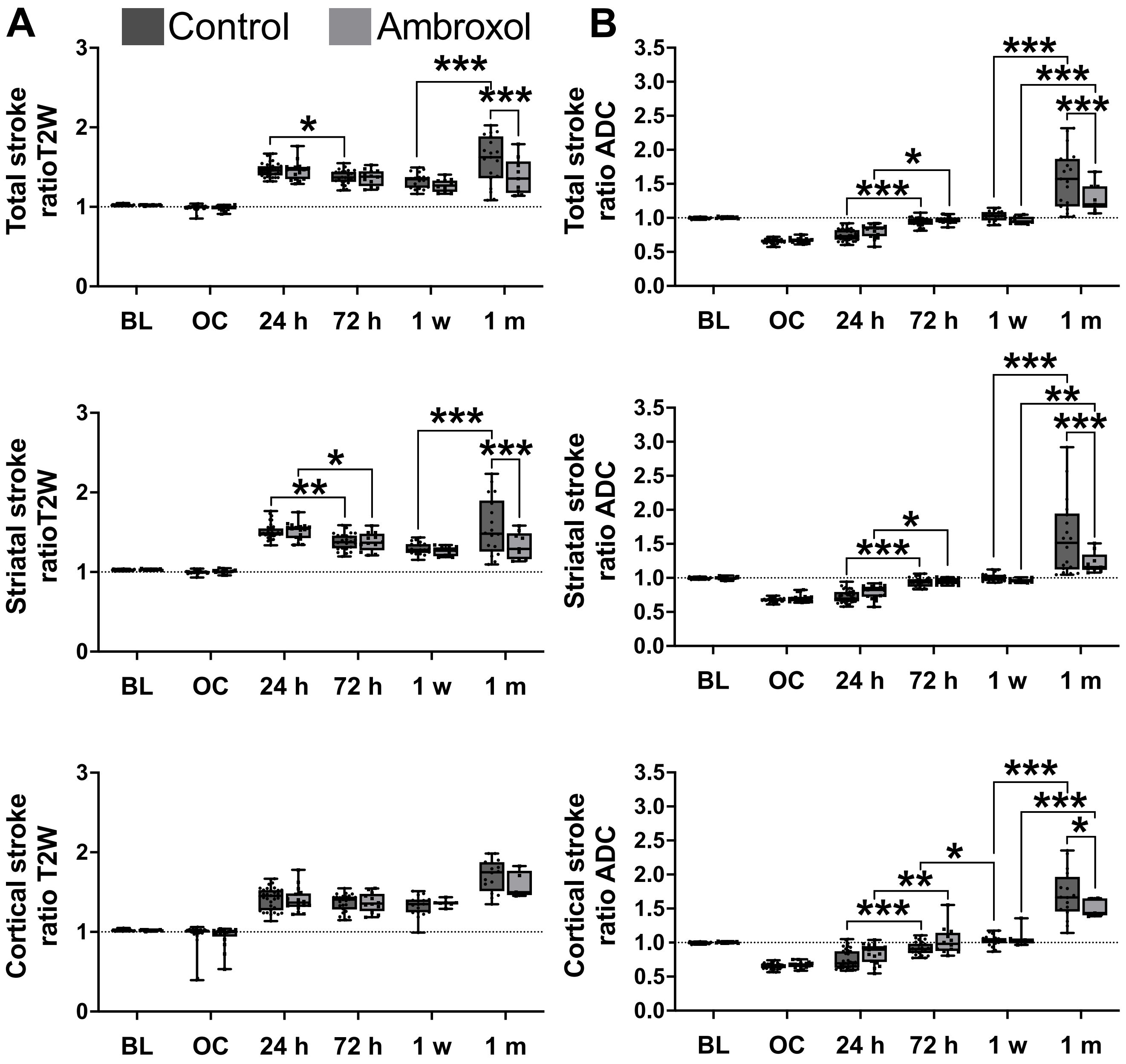

Supplementary Figure 1: Ratio of ADC and T2 values between ipsi- and contralesional hemisphere. Quantified values for the ratio between the ipsi- and contralesional **(A)** T2 and **(B)** ADC over time for combined regions of striatum and cortex, only striatum and only cortex are shown in boxplot graphs. 1 refers to no change between hemispheres, values higher mean higher values for the ipsilesional hemisphere, lower values mean lower values for the ipsilesional hemisphere compared to contralesional. Each boxplot shows the median value (central line) and the 25th to 75th percentiles. Whiskers represent the minimum and maximum values. Rm-ANOVA was performed for statistical analysis, followed by Fischer's test. Rm-ANOVA results are presented in Supplementary Table 4 index no. 7-9 (ipsi/contra T2W ratio) und 13-15 (ipsi/contra ADC ratio). * refers to p<0.05, ** refers to p<0.01 and *** refers to p<0.001. BL (baseline), OC (occlusion).

Histology

The H&E staining (Supplementary Figure 2) in the control animal revealed a large lesion on right hemisphere, affecting both cortex and deep brain, with cavitations and cystic spaces. The infarct area was surrounded by gliosis as seen by the increased number of nucleated cells and the GFAP stain, characteristic of chronic stroke lesions. The cleaved Caspase 3 immune staining revealed focal apoptosis in the outer margins of the lesion. The H&E staining in the Ambroxol brain revealed an extensive damage as well, but without cavitations and cystic structures. Additionally, the glial scar was more prominent as revealed by the GFAP immune staining. Finally, the activated Caspase 3 showed a prominent presence of apoptotic nuclei.

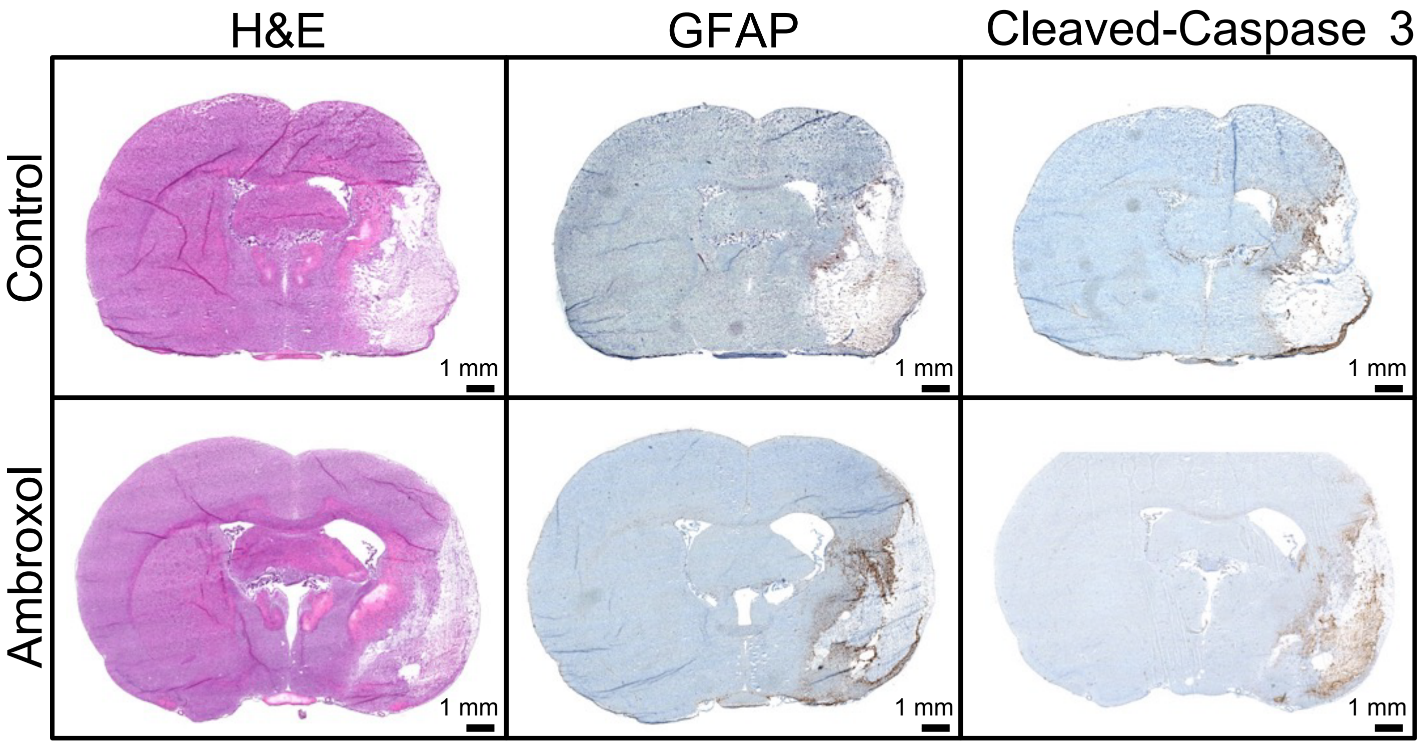

Supplementary Figure 2: Histological assessment of final timepoint. Hematoxylin and eosin (H&E) histology and immunohistochemistry of glial fibrillary acidic protein (GFAP) and cleaved caspase 3 of one control and one Ambroxol treated animal after 1 month.

Behavioral assessments

Post-hoc analysis showed that all animals were able to cross a beam equally at baseline (Fig. 3A). However, at 24 h the outcome of the control group significantly worsened to a score of 3.08 ± 2.23, while the Ambroxol group presented an average improved score of 1.92 ± 2.03. No significant difference was found at any other time point. A normalized success rate analysis mirrored the outcome of the beam walk score results (Fig. 3B). At baseline, all animals were able to cross the beam, at 24 h an inverse peak showed a distinct decline of the performance, which recovered relatively quickly at 72 h for both groups. The results demonstrate significantly worse motor-sensory and coordination deficits in the control animals at 24 h.

In the sticky label test animals required an increased time to remove the adhesive when comparing the values at baseline and 24 h post-stroke. Removal latency decreased after 24 h steadily over the time points. Ambroxol-treated animals removed the sticky label significantly faster (63.14 ± 47.35 s) than control treated animals (105.29 ± 32.45 s) at 24 h post-stroke (Fig. 3C). Interestingly, Ambroxol animals returned to baseline values already after 1 week, while control animals reached baseline removal-time only after 1 month. The successful tape removals were also quantified, which showed an inverse curve progression to the removal time (Fig. 3D). In summary, the sensory deficits assessed through sticky label test were significantly worse at 24 and 72 h in Control animals than in Ambroxol animals.

We further examined motor function directly using the grip test (Fig. 3E). Here the animal's paws are placed on a grid and they are gently pulled back. The tension required for animals to release the grid is directly measured providing an objective measure of limb strength. Here, post-hoc analysis showed a significant difference between the groups at 1 month after stroke induction. Interestingly, there were no other statistical differences between the groups at 24 h, 72 h and 1-week post-stroke. Finally, the Open field test, which measures animal anxiety and movement yielded no significant differences between the groups (Fig. 3F).

Taken all together, there were significantly less sensory, motor and coordination deficits found on the Ambroxol animals in comparison to control animals starting at 24 h after stroke on set.

Fractional anisotropy (FA) T-test maps

Spatially normalizing the imaging datasets allowed us to calculate fairly reliable voxel-wise t-test images between the control and Ambroxol groups independently for each time point. White voxels on the combined images in supplementary Supplementary Figure 3A represent a statistically significant difference of $p < 0.05$ between the two groups for that specific voxel. Significant voxel quantification normalized by region is plotted in supplementary Supplementary Figure 3B.

We first looked at the normal distribution of voxel-wise significant voxels between the groups in the baseline situation, which showed no relevant or increased differences prior to stroke induction. Beginning at 24 h however, this voxel-wise analysis confirmed a larger number of significant voxels with white matter degeneration in control animals compared to Ambroxol. The quantified number of significant voxels can be seen in supplementary Supplementary Figure 3B, which shows the increased degeneration in the external capsule, the internal capsule and genu. Significant differences between the groups were found at 24 h in the internal capsule and the genu, while most significant voxels were found in the external capsule at 72 h and 1 week. Differences between the groups reduced at 1 month post stroke induction, similar to the VOI analysis. The graph shows, the proportion of significant voxels per brain region between the groups. It shows that at 24 h post stroke induction, 35% of the volume of the ipsilesional internal capsule has significantly increased FA in comparison to Ambroxol animals. However, FA continues to decline in the control group up to the 1-week time point, while the Ambroxol animals maintain white matter integrity with no further changes. VOI-based analysis confirmed this pattern over time showing significant differences at 24 h and 1 week (Fig. 4D). Significances increase in the external capsule increases at 72 h until 1 week so that more than 50% of the volume of this region is significantly different between both groups, similarly to absolute FA values (Fig. 4C). The significant volume differences in the genu was similar in both hemispheres, starting at around 10% at 24 h and increasing steadily until 1 week up to 23% on the ipsilesional and 19% on the contralesional hemisphere. All other contralesional VOIs had similar significant differences between 2% and 14%. All significant differences reduced on both hemispheres closer to baseline at 1 month post stroke, denoting a normalization of white matter edema over time.

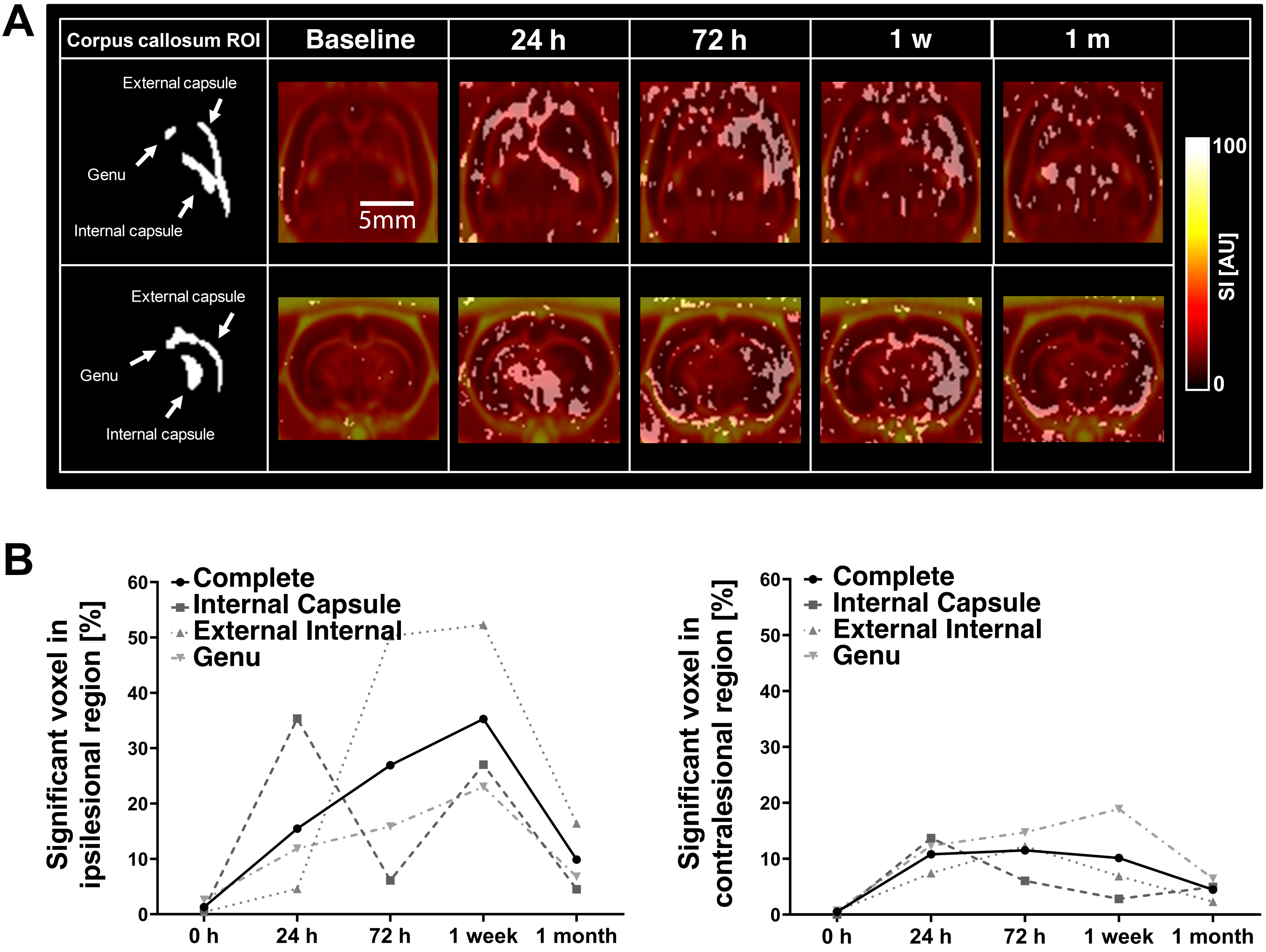

Supplementary Figure 3: Specific white matter degeneration evaluated voxel-wise over time. T-test maps were generated by comparing the calculated FA maps of the control and Ambroxol animals at different time points. **(A)** The upper panel shows exemplary volumes of interest (VOI) of the corpus callosum. The left panel shows the average FA images of all evaluated animals with its overlaying t-test map for every time point. White voxels of the overlay represent a significant difference between both groups of $p < 0.05$. **(B)** The quantifications of the significant voxels per region are shown for both hemispheres. OC (occlusion), SI (signal intensity).

Functional connectivity

Supplementary Figure 4 and 5 show functional connectivity data on a whole brain level. The median Pearson's r was 0.42 for the control group and 0.39 for the Ambroxol group at baseline. The overall Pearson's r reduced for both groups at 24 h post stroke (Control: r = 0.36; Ambroxol: r = 0.25). While the median Pearson's r recovered for Ambroxol animals back to r = 0.32, connectivity for control animals remained at r = 0.34. For both groups, overall connectivity did not reach baseline values again.

Highest network-average correlations were found at baseline for the motor pathways with a Pearson's r = 0.56 for the control group and a Pearson's r = 0.51 for the Ambroxol group (Fig. 5B). There were no significant differences between the motor network connections of both groups before stroke induction and therapy administration.

Fig. 6 in the main manuscript shows the changes in connectivity limited to the motor network. The biggest reduction of the z-transformed Pearson's r value was found in both groups between the ipsi- and contralesional striatum at 24 h. Pearson's r dropped 51% from r = 1.10 to r = 0.54 for the control group and even 55% r = 1.04 to r = 0.47 for the Ambroxol group. As shown in Fig. 6 there was one additional significant reduced rs-FC in the Ambroxol group after FDR correction from baseline to 24 h: the correlation between the ipsilesional S1 cortex and the contralesional striatum. Control animals presented various significant reduced rs-FC from baseline to 24 h: the correlations between the ipsi- and contralesional M1 cortex, S1 cortex and thalamus as well as the correlations between the contralesional S1 cortex and ipsilesional M1 cortex.

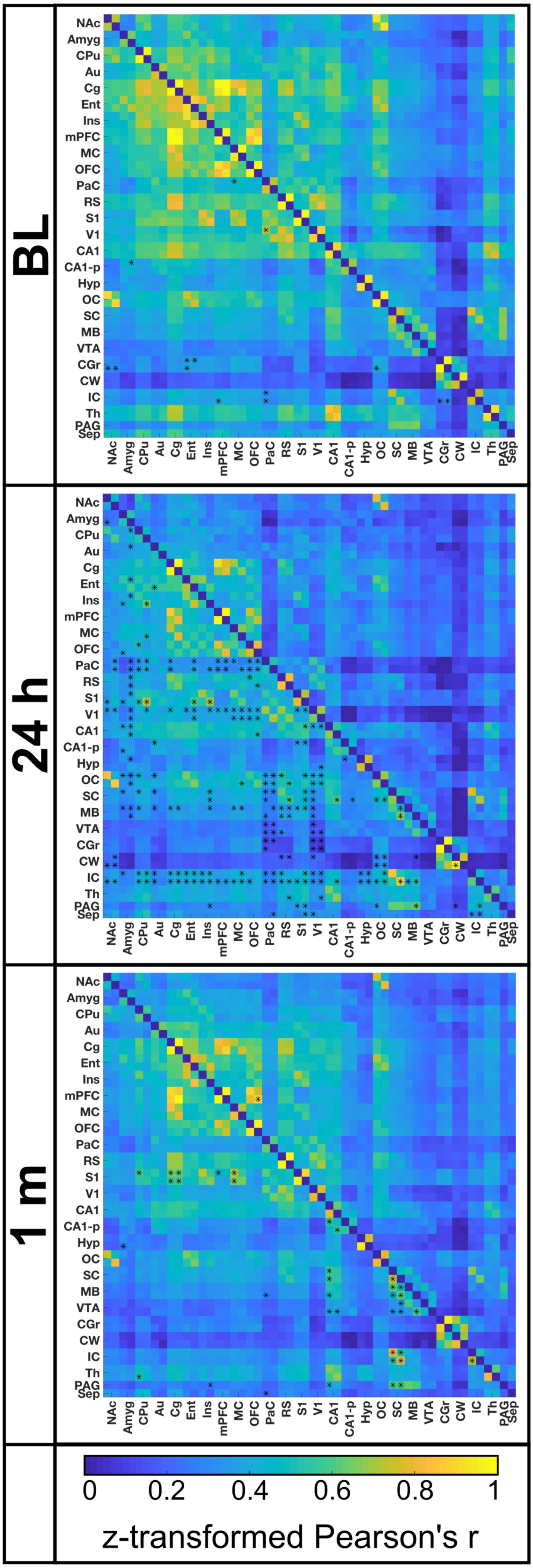

Supplementary Figure 4: Resting-state functional connectivity between the groups in whole brain. Pair-wise rs-FC of regions belonging to the motor network are shown as a heat map with strongest correlations in yellow and weakest in blue (z-transformed Pearson's r). The diagonal line shows self-correlations which are set to zero (dark blue). The lower triangle represents the connectivity of the control group and the upper triangle represents the connectivity of the Ambroxol group. Connectivity maps between the groups at baseline and 24 h and 1 month post stroke induction for the whole brain is shown. Control group: n = 17; Ambroxol group: n = 9. Statistics from two-way repeated measures ANOVA. Significances found are shown with an asterisk referring to a statistically significant higher connectivity in the respective group at $p < 0.05$ without FDR correction. Abbreviations for brain regions can be found in Supplementary Table 7. BL (baseline).

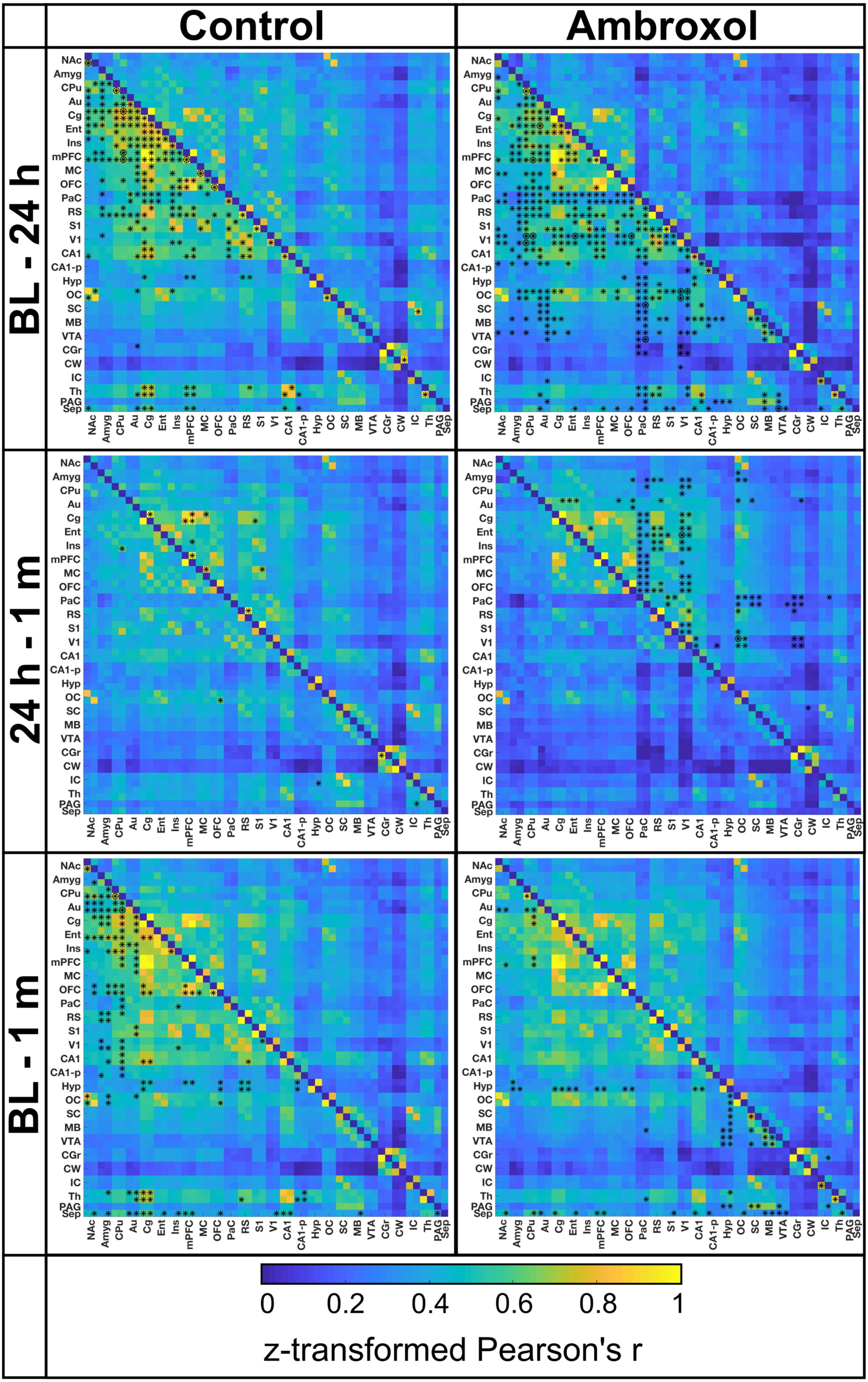

Control
Ambroxol
BL - 24 h
24 h - 1 m
BL - 1 m
NAc
Amyg
CPu
Au
Cg
Ent
Ins
mPFC
MC
OFC
PaC
RS
S1
V1
CA1
CA1-p
Hyp
OC
SC
MB
VTA
CGr
CW
IC
Th
PAG
Sep
0
0.2
0.4
0.6
0.8
1
z-transformed Pearson's r

Supplementary Figure 5: Resting-state functional connectivity of the whole brain compared for both groups between time points. The diagonal line shows self-correlations which are set to zero (dark blue). The lower triangle represents the connectivity of the first time point (titled fist) and the upper triangle represents the second time point (titled second). Connectivity maps at baseline to 24 h, 24 h to 1 month and baseline to 1 month for the whole brain are shown. Control group: n = 17; Ambroxol group: n = 9. Different time points were analyzed using two-way repeated measures ANOVA followed by FDR correction. Significances found by the ANOVA are marked with an *, referring to a significant higher connectivity with a p-value < 0.05 for the respective time point before FDR correction. Circled asterisks remained significant after FDR correction. Abbreviations for brain regions can be found in Supplementary Table 7. BL (baseline).

References

1. Longa EZ, Weinstein PR, Carlson S, Cummins R. Reversible middle cerebral artery occlusion without craniectomy in rats. *Stroke*. 1989;20(1):84-91. doi:10.1161/01.STR.20.1.84

2. Castaneda-Vega S, Katiyar P, Russo F, et al. Machine learning identifies stroke features between species. *Theranostics*. 2021;11(6):3017-3034. doi:10.7150/thno.51887

3. Schiffer WK, Mirrione MM, Biegon A, Alexoff DL, Patel V, Dewey SL. Serial microPET measures of the metabolic reaction to a microdialysis probe implant. *J Neurosci Methods*. 2006;155(2):272-284. doi:10.1016/j.jneumeth.2006.01.027

4. Chuang KH, Lee HL, Li Z, et al. Evaluation of nuisance removal for functional MRI of rodent brain. *Neuroimage*. 2019;188:694-709. doi:10.1016/j.neuroimage.2018.12.048

5. Wehrl HF, Hossain M, Lankes K, et al. Simultaneous PET-MRI reveals brain function in activated and resting state on metabolic, hemodynamic and multiple temporal scales. *Nat Med*. 2013;19(9):1184-1189. doi:10.1038/nm.3290

6. Paxinos G, Watson C. *The Rat Brain in Stereotaxic Coordinates*. Academic Press Inc.; 1998.

7. Brett M, Anton JL, Valabregue R, Poline JB. Region of interest analysis using an SPM toolbox [abstract]. Presented at the 8th International Conferance on Functional Mapping of the Human Brain, June 2-6, 2002, Sendai, Japan. Available on CD-ROM in NeuroImage, Vol 16, No 2, abstract 497. 16.

8. Dieterle F, Ross A, Schlotterbeck G, Senn H. Probabilistic quotient normalization as robust method to account for dilution of complex biological mixtures. Application in 1H NMR metabonomics. *Anal Chem*. 2006;78(13):4281-4290. doi:10.1021/AC051632C

9. Warach S, Gaa J, Siewert B, Wielopolski P, Edelman RR. Acute human stroke studied by whole brain echo planar diffusion-weighted magnetic resonance imaging. *Ann Neurol*. 1995;37(2):231-241. doi:10.1002/ana.410370214

Supplementary Tables:

Supplementary Table 1: Total animal number for imaging data for each time point

Time point	SV, ADC, T2W, FA data		fMRI data	
	Control	Ambroxol	Control	Ambroxol
**BL**	36	17	17	9
**OC**	36	17	-	-
**24 h**	36	17	17	9
**72 h**	26	12	-	-
**1 w**	19	10	-	-
**1 m**	19	10	17	9

Abbreviations: SV (stroke volume), ADC (apparent diffusion coefficient), FA (fractional anisotropy), fMRI (functional MRI), BL (baseline), OC (occlusion).

Supplementary Table 2: Total animal number for all behavioral tests for each time point

Time point	Beam walk		Sticky label		Open field		Grip test		Total available	
	Control	Ambroxol	Control	Ambroxol	Control	Ambroxol	Control	Ambroxol	Control	Ambroxol
**BL**	32	15	31	14	22	11	33	15	33	15
**24 h**	33	15	31	14	21	10	33	15	33	15
**72 h**	26	12	25	11	18	10	26	12	26	12
**1 w**	19	10	18	9	17	9	19	10	19	10
**1 m**	19	10	18	9	12	8	19	10	19	10

The total number of animals available for behavioral evaluation per time point are shown on the last columns on the right. Multiple animals in the open field were excluded due to technical software failure. Three animals from the sticky label test were excluded since they were not able to remove the sticky tape at any time point. 1 animal from the beam walk test got removed from the analysis at baseline because it did not complete any of the 5 iterations.
Abbreviations: BL (baseline).

Supplementary Table 3: Results of repeated measures Analysis of Variance (rm-ANOVA) on multiple parameters confirming the similarity between the control groups NaCl and PEG.

No.	Method	Parameter	Region	Variable	df	df-error	F-value	p-value
1	MRI	Stroke volume	Whole stroke	Group	1	92	0.18	0.68
				Group*Time	3	92	0.18	0.91
			Striatum stroke	Group	1	92	0.85	0.36
				Group*Time	3	92	0.07	0.98
			Cortex stroke	Group	1	92	0.21	0.65
				Group*Time	3	92	0.17	0.92
2	MRI	ADC	Whole stroke	Group	1	92	0.03	0.87
				Group*Time	3	92	0.01	0.99
			Striatum stroke	Group	1	91	0.01	0.90
				Group*Time	3	91	0.24	0.86
			Cortex stroke	Group	1	88	0.50	0.48
				Group*Time	3	88	1.11	0.35
3	MRI	T2	Whole stroke	Group	1	92	0.02	0.89
				Group*Time	3	92	0.69	0.55
			Striatum stroke	Group	1	91	0.05	0.81
				Group*Time	3	91	0.55	0.64
			Cortex stroke	Group	1	88	0.42	0.51
				Group*Time	3	88	1.02	0.39
4	MRI	White matter integrity FA	Capsula interna	Group	1	92	0.04	0.85
				Group*Time	3	92	0.43	0.73
			Genu	Group	1	92	0.15	0.69
				Group*Time	3	92	0.20	0.89
			Capsula externa	Group	1	92	0.04	0.83
				Group*Time	3	92	0.07	0.97
5	Behavior	Beam walk – score	-	Group	1	89	0.04	0.84
				Group*Time	3	89	0.07	0.97
		Sticky label – time (s)	-	Group	1	84	0.03	0.87
				Group*Time	3	84	0.75	0.52
		Grip test – recovery (%)	-	Group	1	89	5.9	0.02
				Group*Time	3	89	1.1	0.34
		Open field – distance (m)	-	Group	1	60	0.27	0.60
				Group*Time	3	60	0.3	0.82

Abbreviations: df (degrees of freedom), F (F-value), p (p-value). Variable 'Group' refers to the comparison of NaCl and PEG. Group*Time refers to the interaction of group and time. All evaluations performed with repeated measures ANOVA.

Supplementary Table 4: Results of specific statistical parameters between Ambroxol and control group by repeated measures Analysis of Variance (rm-ANOVA)

No.	Method	Parameter	Stroke region	Variable	df	df-error	F-value	p-value
1	MRI	Stroke volume	Whole stroke	Group	1	141	10.39	0.002
				Time	3	141	7.58	<0.001
				Group*Time	3	141	0.11	0.951
2	MRI	Stroke volume	Striatum stroke	Group	1	141	22.56	<0.001
				Time	3	141	30.45	<0.001
				Group*Time	3	141	0.36	0.784
3	MRI	Stroke volume	Cortex stroke	Group	1	141	10.22	0.002
				Time	3	141	3.11	0.028
				Group*Time	3	141	0.07	0.977
4	MRI	T2W	Whole stroke	Group	1	140	6.2	0.014
				Time	3	140	6.46	<0.001
				Group*Time	3	140	2.74	0.046
5	MRI	T2W	Striatum stroke	Group	1	138	6.91	0.01
				Time	3	138	14.61	<0.001
				Group*Time	3	138	2.98	0.034
6	MRI	T2W	Cortex stroke	Group	1	129	0.65	0.42
				Time	3	129	2.39	0.072
				Group*Time	3	129	1.26	0.291
7	MRI	Ipsi/contra T2W ratio	Whole stroke	Group	1	140	6.63	0.011
				Time	3	140	9.43	<0.001
				Group*Time	3	140	2.7	0.048
8	MRI	Ipsi/contra T2W ratio	Striatum stroke	Group	1	138	5.28	0.023
				Time	3	138	12.65	<0.001
				Group*Time	3	138	3.38	0.02
9	MRI	Ipsi/contra T2W ratio	Cortex stroke	Group	1	129	0.9	0.346
				Time	3	129	17.1	<0.001
				Group*Time	3	129	0.84	0.472
10	MRI	ADC	Whole stroke	Group	1	140	5.32	0.023
				Time	3	140	82.62	<0.001
				Group*Time	3	140	6.44	<0.001
11	MRI	ADC	Striatum stroke	Group	1	138	5.56	0.02
				Time	3	138	49.74	<0.001
				Group*Time	3	138	6.16	<0.001
12	MRI	ADC	Cortex stroke	Group	1	129	0.06	0.803
				Time	3	129	91.28	<0.001
				Group*Time	3	129	3.33	0.022
13	MRI	Ipsi/contra ADC ratio	Whole stroke	Group	1	140	4.3	0.04
				Time	3	140	77.65	<0.001
				Group*Time	3	140	5.85	<0.001
14	MRI	Ipsi/contra ADC ratio	Striatum stroke	Group	1	138	5.56	0.028
				Time	3	138	49.74	<0.001
				Group*Time	3	138	6.16	<0.001
			Cortex stroke	Group	1	129	0.06	0.684

15	MRI	Ipsi/contra ADC ratio		Time	3	129	91.28	<0.001
				Group*Time	3	129	3.33	0.031
16	Behavior	Beam walk – score	-	Group	1	136	4.47	0.036
			-	Time	3	136	4.73	0.004
			-	Group*Time	3	136	0.73	0.538
17	Behavior	Sticky label – time (s)	-	Group	1	127	12.16	<0.001
			-	Time	3	127	13.43	<0.001
			-	Group*Time	3	127	0.86	0.462
18	Behavior	Grip test – recovery (%)	-	Group	1	136	4.53	0.035
			-	Time	3	136	4.37	0.006
			-	Group*Time	3	136	1.74	0.163
19	Behavior	Open field – distance (m)	-	Group	1	97	1.66	0.201
			-	Time	3	97	1.17	0.325
			-	Group*Time	3	97	0.85	0.471
20	MRI	White matter integrity FA	Whole corpus callosum	Group	1	141	4.2	0.042
				Time	3	141	1.64	0.184
				Group*Time	3	141	4.32	0.006
21	MRI	White matter integrity FA	Internal capsule	Group	1	141	1.2	0.275
				Time	3	141	2.82	0.041
				Group*Time	3	141	4.58	0.004
22	MRI	White matter integrity FA	External capsule	Group	1	141	14.69	<0.001
				Time	3	141	3.59	0.015
				Group*Time	3	141	4.21	0.007
23	MRI	White matter integrity FA	Genu	Group	1	141	0.76	0.386
				Time	3	141	1.93	0.127
				Group*Time	3	141	4.35	0.006

Abbreviations: df (degrees of freedom), ADC (apparent diffusion coefficient) FA (fractional anisotropy), CC (corpus callosum), Internal (internal capsule), External (external capsule). Variable 'Group' refers to the comparison of Ambroxol and combined control group; Time refers to the evaluation of timepoint analysis and Group*Time to the interaction evaluation.

Supplementary Table 5: Complete comparison of Ambroxol and Control metabolomic brain data in average fold changes (FC, log_2 scale) in the contralesional and ipsilesional cortex and striatum (A-D).

Description	Metabolite	Cortex				Striatum			
		Contra		Ipsi		Contra		Ipsi	
			log_2FC		log_2FC		log_2FC		log_2FC
**Energy metabolism**	Adenosine	↓	1.0	↓	0.4	↓	0.2	↓	0.6
	Adenosine monophosphate (AMP)	↑*	**0.4**	↓	0.2	↑	0.1	↓	0.4
	Adenosine triphosphate (ATP)	↑	0.2	↓	0.1	↓	0.9	↑	0.5
	Inosine	↑	0.2	↓	0.1	↓	0.9	↓	0.4
	Inosine monophosphate (IMP)	↓	0.5	↓	1.5	↓	0.6	↓	0.2
	Nicotinamide adenine dinucleotide (NAD)	↑*	**1.0**	↑	0.3	↑	0.5	↑	0.3
	Creatine	↑	0.2	↓	0.2	≈	0.0	↓	0.1
	Phosphocreatine	↑	1.9	↑	1.2	↑	0.5	↑*	**1.7**
**Vitamins**	Ascorbate (vitamin C)	↑	0.8	↓	2.4	↑	0.4	↑	0.6
	Pantothenate (vitamin B5)	↓	1.5	↑*	**1.4**	↑	0.3	↑*	**1.0**
**Glycolysis**	Glucose	↓	1.0	↑	1.0	↓*	**1.1**	↑	0.7
	Lactate	↑	0.1	↑	0.1	↓	0.1	↓*	**0.3**
**Krebs Cycle (TCA)**	Citrate	↓	1.0	↑	0.1	↓*	**1.1**	↑	0.7
	Succinate	↑	0.1	↓	0.1	≈	0.0	↓	0.3
**Short chain fatty acids**	Acetate	↓*	**1.1**	≈	0.0	↓*	**0.9**	↓	0.3
	Formate	↓	0.6	↓	0.2	↓	0.2	↓	0.3
**Osmolytes**	Beta-alanine	N/A	N/A	N/A	N/A	↓	0.9	≈	0.0
	Glycerol	↓	0.7	≈	0.0	↓	1.1	↓	0.7
	Taurine	↑	0.2	↓	0.1	↑	0.1	≈	0.0
	scyllo-Inositol	↑	0.3	≈	0.0	↓	0.4	↑	0.3
**Neuronal tissue-associated metabolites**	gamma-Aminobutyrate (GABA)	≈	0.0	↑	0.1	↓	0.3	↓	0.5
	N-Acetylaspartate (NAA)	↑	0.2	↓	0.2	↓	0.1	↓	0.2
**Branched-chain amino acids (BCAAs)**	Isoleucine	↑	0.9	↑	0.9	↑	0.4	↑	0.3
	Leucine	↑	0.7	↑	0.6	↓	0.1	↓	0.1
	Valine	↑*	**0.8**	↑*	**0.8**	↑	0.2	≈	0.0
**Amino acid metabolism**	Alanine	≈	0.0	↑	0.1	≈	0.0	↓	0.3
	Aspartate	↑*	**0.4**	≈	0.0	≈	0.0	↓	0.2
	Glutamate	↑	0.2	↓	0.2	≈	0.0	↓	0.1
	Glutamine	↑	0.1	↓	0.1	≈	0.0	↑	0.2
	3-Hydroxyisovalerate (3-HIV)	↓	0.6	↑	0.4	↓	0.8	≈	0.0
	Lysine	↓	1.0	↑	0.3	↓*	**1.1**	↑	0.3
	Phenylalanine	↓	0.6	↑	1.1	↓	0.2	↑	0.5
	Tyrosine	↑	0.7	↑*	**1.3**	↑	0.5	↑	0.5

**One carbon metabolism**	Glycine	↑	0.3	↑	0.4	≈	0.0	↓	0.1
	Guanidinoacetate (GuAc)	↓	0.5	↑	0.9	↑	0.4	↓	0.2
	Serine	↑	0.5	≈	0.0	≈	0.0	↓	0.2
	Threonate	↑	0.4	↑	0.5	↓	0.1	↑	0.6
	Threonine	↑	0.5	↑	0.5	↑	0.3	≈	0.0
**Ketone bodies**	Acetone	↓	0.2	↑*	**1.7**	↓*	**1.1**	↑	0.9
	3-Hydroxybutyrate (3-HB)	↑	0.4	↑*	**1.4**	↑	0.1	↑	0.2
**Cell membrane synthesis, Kennedy pathway**	Acetylcholine	↑*	**0.8**	↓	0.7	↑	0.2	≈	0.0
	Choline	↓*	**0.6**	↑	0.7	↓	0.6	↑	0.1
	Ethanolamine	↑	0.9	↓	0.5	↓	0.1	↓*	**1.1**
	Glycerophosphocholine	↑	0.4	↑	0.1	↑	0.1	↑	0.1
	myo-Inositol	↑	0.1	↓	0.1	≈	0.0	↓	0.4
	Phosphocholine (PCh)	↑	0.3	↑	0.2	≈	0.0	≈	0.0
**Redox metabolism**	Glutathione	↑*	**0.9**	↑	1.5	↓	0.6	↑*	**1.3**
	Reduced glutathione (GSSG)	↓	0.1	↓	0.2	↓	0.1	↑	0.5

Abbreviations: AMP (adenosine monophosphate), NAD (nicotinamide adenine dinucleotide), Contra (contralesional), Ipsi (ipsilesional). T-test comparisons were made between the control and Ambroxol groups, * p-value < 0.05. Results not significant after FDR.

Supplementary Table 6: Metadata considered in metabolomics heatmap data analysis (Fig. 8). Stroke volume values per each animal that was submitted for metabolomics study.

Group	Rat	Stroke volumes [cm^3]
Ambroxol	R0	0.424
	R1	0.424
	R2	0.120
	R3	0.486
Control	R4	0.263
	R5	0.404
	R6	0.307
	R7	0.256
	R8	0.242

Supplementary Table 7: All 52 brain regions used for fMRI correlations including ROI volume and abbreviation

Brain region (ROI)	Hemisphere	ROI volume [cm^3]	Position on correlation matrix	Abbreviation
Nucleus Accumbens	left	0.008	1	NAc
	right		2	
Amygdala	left	0.021	3	Amyg
	right		4	
Caudate Putamen	left	0.044	5	CPu
	right		6	
Auditory Cortex	left	0.028	7	Au
	right		8	
Cingulate Cortex	left	0.014	9	Cg
	right		10	
Entorhinal Cortex	left	0.059	11	Ent
	right		12	
Insular Cortex	left	0.021	13	Ins
	right		14	
Medial Prefrontal Cortex	left	0.006	15	mPFC
	right		16	
Motor Cortex	left	0.033	17	MC
	right		18	
Orbitofrontal Cortex	left	0.019	19	OFC
	right		20	

Parietal Cortex	left	0.008	21	PaC
	right		22	
Retrosplenial Cortex	left	0.019	23	RS
	right		24	
Somatosensory Cortex	left	0.072	25	S1
	right		26	
Visual Cortex	left	0.036	27	V1
	right		28	
Anterodorsal Hippocampus	left	0.025	29	CA1
	right		30	
Posterior Hippocampus	left	0.010	31	CA1-p
	right		32	
Hypothalamus	left	0.018	33	Hyp
	right		34	
Olfactory Cortex	left	0.014	35	OC
	right		36	
Superior Colliculus	left	0.007	37	SC
	right		38	
Midbrain	left	0.011	39	MB
	right		40	
Ventral Tegmental Area	left	0.006	41	VTA
	right		42	
Cerebellum – grey matter	left	0.075	43	CG
	right		44	
Cerebellum – white mater	left	0.023	45	CW
	right		46	
Inferior Colliculus	left	0.006	47	IC
	right		48	
Thalamus	left	0.031	49	Th
	right		50	
Periaqueaductal Gray	-	0.010	51	PAG
Septum	-	0.009	52	Sep
